# Synthesis of phosphatidic acids *via* cobalt(salen) catalyzed epoxide ring-opening with dibenzyl phosphate†

**DOI:** 10.1039/d2ob00168c

**Published:** 2022-03-03

**Authors:** Ruben L. H. Andringa, Marijn Jonker, Adriaan J. Minnaard

**Affiliations:** Stratingh Institute for Chemistry, University of Groningen Nijenborgh 7 9747AG Groningen The Netherlands a.j.minnaard@rug.nl

## Abstract

With a CoIII(salen)OTs catalyst, dibenzyl phosphate ring-opens a variety of terminal epoxides with excellent regio-selectively and yields up to 85%. The reaction is used in a highly efficient synthesis of enantiopure mixed-diacyl phosphatidic acids, including a photoswitchable phosphatidic acid mimic.

## Abstract 2

A cobalt-catalyzed, phosphate-induced, ring-opening reaction is used in an efficient synthesis of enantiopure mixed-diacyl phosphatidic acids, including a photoswitchable phosphatidic acid mimic.

## Graphical abstract

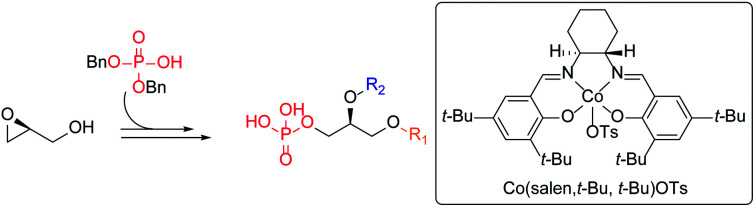

Phosphatidic acids (phosphorylated diacylglycerols) form a diverse and vital class of compounds in both prokaryotes and eukaryotes.^1–3^ Although a minor fraction of the lipid membrane, phosphatidic acids are important for cell signaling and interact with various enzymes modulating their activity.^4,5^ Furthermore, phosphatidic acids are key intermediates in glycerophospholipid biosynthesis.^6^

In chemical biology, photo-switchable phospholipids have been developed^7,8^ to study and manipulate membrane function. Tei *et al.* have shown very recently that it is possible to control phosphatidic acid signaling, specifically mTOR and Hippo signaling, employing light-switchable phosphatidic acids.^9^

The stereoselective synthesis of diacyl-glycerols, the overarching family comprising phosphatidic acids, with identical acyl residues is relatively straightforward. However, the synthesis of mixed-diacylglycerols, bearing two different acyl residues, is considerably more challenging.^10–19^ This is partly due to a facile “acyl-shift”; an intramolecular *trans*-esterification reaction. An acyl residue on the secondary position will readily shift to a free primary hydroxyl group as it experiences less steric hindrance.^20,21^ This isomerization is catalyzed by traces of (Lewis) acids and bases, also those present in silica and aluminum oxide, making synthesis and purification of these compounds problematic. This problem is worsened by the difficulty to separate the produced mono-acyl glycerols and the near impossibility to separate regio-isomeric diacylglycerols and phosphatidic acids. Older synthetic strategies for diacylglycerols and phosphatidic acids^10–19^ generally avoid acyl shift by using orthogonal protecting groups, which is not beneficial for step-count and atom efficiency.

A more efficient strategy is to regio-selectively open a terminal epoxide to install a fatty ester.^15–19^ This ring-opening strategy effectively avoids the need for orthogonal protecting groups, making the synthesis shorter. Currently, the most efficient strategies for the synthesis of phosphatidic acids have been reported by the group of Konradsson and our group (Fig. 2, 5–8).

Konradsson treats enantiopure glycidol with dibenzylphosphoramidite,^15^ followed by oxidation, and then installs a long-chain fatty acid in a Lewis acid-mediated regio-selective ring opening. The resulting product is subsequently converted into either the mono-acyl phosphatidic acid or a mixed-di-acyl phosphatidic acid. Primary hydroxy groups are effectively avoided in this route, and acyl shift from a primary to a secondary position is considerably slower than *vice versa*. In addition, the bulky phosphate group attenuates this acyl shift even more, so this approach affords pure mixed-acyl glycerophosphates.

In 2016, our group reported an even more efficient regio-selective epoxide ring-opening esterification employing Co(salen) catalyst I.^22^ Immediate subsequent esterification provided the desired protected hetero-di-acyl glycerols in excellent yields (Fig. 2, 9–12). A “near-instantaneous” deprotection protocol of the TBS group with BF_3_·CH_3_CN followed by neutral quench, avoided acyl shift and produced pure 1,2-diacylglycerols. These were further converted into the desired glycerophosphates employing a phosphoramidite coupling followed by oxidation. It was shown later that this cobalt catalyzed epoxide-opening esterification could also be used for the synthesis of enantiopure triacylglycerols.^23^

As illustrated, the synthesis of phosphatidic acids has seen significant improvements over the years, but the routes still are quite laborious and rely on moisture-sensitive phosphoramidite coupling reactions. To improve on these strategies, a switch from a trivalent phosphorus source to a pentavalent phosphorus source would be advantageous. Switching from P(iii) to P(v) can be very advantageous as shown recently for (solid phase) oligonucleotide synthesis.^24^ Grosdemange-Billiard and co-workers reported the Lewis acid mediated ring-opening of racemic benzyl glycidol with dibenzyl phosphate (Fig. 2, 13–14).^25^ In a screening of a multitude of metal salts, the best results were obtained with a stoichiometric amount of CuI. The method was used for a straightforward synthesis of dihydroxyacetone phosphate.

Inspired by the work of Grosdemange-Billiard and our cobalt catalyzed epoxide ring-opening esterification (Fig. 2, 9–12), we envisioned the direct installment of a phosphate on the terminal position of glycerol *via* a cobalt catalyzed epoxide ring-opening. This would give straightforward access to enantiopure phosphatidic acids, as a range of enantiopure, protected glycidols is commercially available, and affordable, making them very useful as building blocks for asymmetric synthesis. Co(iii)–salen cat-I was selected for the introduction of a dibenzylphosphate group (as P(v) source) on a terminal epoxide (Fig. 1, 1–4). This catalyst is well-known for the kinetic resolution of terminal epoxides by ring-opening with water, developed by the group of Jacobsen.^26^ In addition, it has been shown to catalyze the nucleophilic attack of a range of alcohols,^27,28^ and acids.^22^ The use of phosphate as a nucleophile has not been reported.

**Fig. 1:**
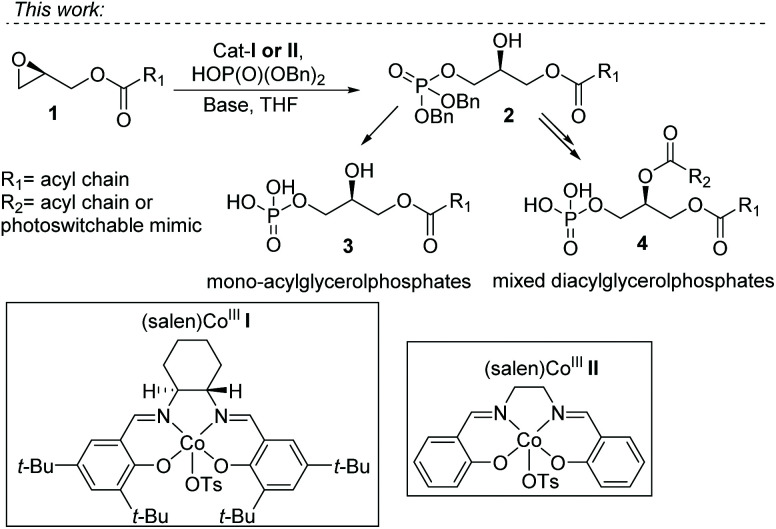
Co^III^-Catalyzed phosphorylation strategy for the synthesis of mono- and mixed-diacyl phosphatidic acids.

**Fig. 2:**
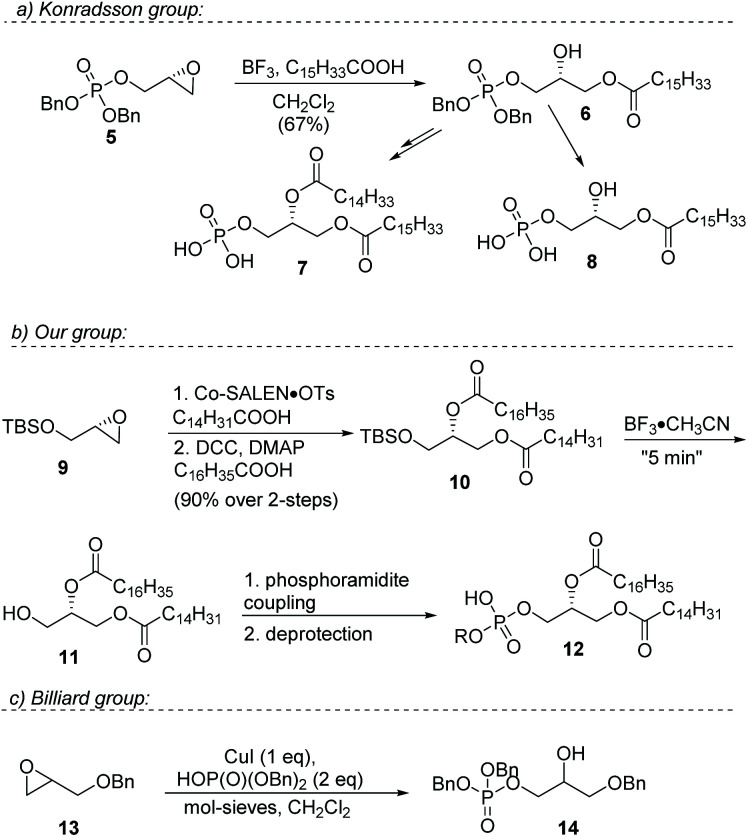
Previous work on the synthesis of mono- and di-acylglycerolphosphates by the Konradsson, the Grosdemange-Billiard, and our group.

With this strategy we effectively block the primary position for 1,2 acyl shift. Thereby we do not require a protecting group on the glycerol backbone. This approach can be used for the synthesis of enantiopure mono- and di-acyl phosphatidic acids. Furthermore, we can use this method for the synthesis of photoswitchable phospholipid mimics. The choice of the phosphate nucleophile deserves special attention. As free phospholipids are difficult to purify, dibenzylphosphate was selected. This produces protected phospholipids that are readily purified by silica gel chromatography. In the final step, hydrogenolysis with Pd/C and hydrogen removes the benzyl substituents, leaving essentially pure product. In cases in which hydrogenolysis is not compatible with functional groups in the acyl residues, treatment with TMSBr is known as an alternative deprotection strategy.

The use of an OTs counterion in the Co(iii)–salen complex, and not the more commonly used acetate, is essential.^27–29^ This is probably because less coordinating counterions facilitate a stronger epoxide coordination as well as a faster addition of the Co–OP(O)(OR)_2_ complex.^30^ To explore if this approach was valid we used the ring-opening of (*S*)-2-((benzyloxy)methyl)oxirane (15) with Co(iii)–salen catalyst I as a model system (Scheme 1). Initially it was difficult to obtain acceptable yields, stagnating at 60%. Elevated temperatures and using catalytic amounts of base had little effect. Additionally, we observed irreproducibility in the obtained yields. Upon switching to a high purity grade dibenzylphosphate, a significant increase in yield and reproducibility was observed, giving the desired product in 78% yield. Purification of the prepared diisopropylethylammonium dibenzylphosphate salt by column chromatography before use, increased the yield even further to 84%.

**Scheme 1:**
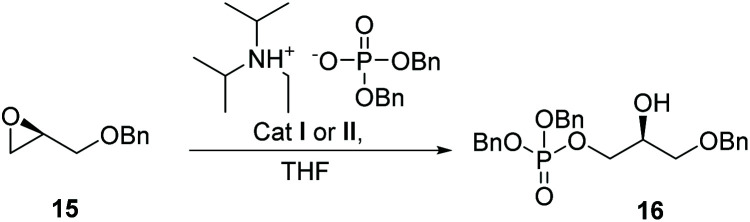
Ring opening phosphorylation.

It is worth mentioning that both the (*R*,*R*) and the (*S*,*S*) enantiomer of I perform similarly for both enantiomers of the glycidol. The optimal reaction conditions comprised 10 mol% of catalyst I, and 1 eq. of diisopropylethylammonium dibenzylphosphate in THF (2 M in benzyl glycidol), providing 84% isolated yield of the desired product (Scheme 1).

Compared to the method of Grosdemange-Billiard,^25^ similar yields are obtained, but using 10% of cobalt catalyst I instead of a stoichiometric amount of Lewis acid, and with just 1 equivalent of dibenzyl phosphate.

The chirality in catalyst I is not used, as the reactions are carried out with enantiopure glycidol derivatives. Therefore, also achiral catalyst II, an even more affordable Co complex, was studied. This catalyst gave good, although consistently somewhat lower, yields (see ESI†) compared to complex I. Chiral catalyst I seems to have a more optimal structure for the ring opening of epoxides, and therefore remained the catalyst of choice.

This first example of a catalyzed ringopening of enantiopure epoxides with a phosphate, avoids the use of phosphoramidite coupling reactions. In addition, it circumvents the necessity of protecting groups on the glycerol backbone. Therefore, we used this finding to develop a very efficient method to prepare mixed diacyl phosphatidic acids.

Phosphorylation of terminal epoxides with the Co(iii)–salen catalyst provides a novel strategy towards mono and mixed-diacyl phosphatidic acids (Scheme 2). (*R*)-Glycidol was first palmitoylated. Subsequently, dibenzyl phosphate was installed on the other primary position employing our optimized conditions, giving the desired product in 68% yield (Scheme 2). With this facile synthesis of mono-acyl glycerolphosphate 25, a range of phosphatic acids can be synthesized. Compound 25 can either be deprotected to give the desired enantiopure mono-acyl phosphatidic acid 32, or can be further substituted on the secondary position. Esterification of the secondary hydroxy group was achieved by straightforward DCC coupling with stearic acid, giving the desired diacyl-glycerol 33 in 68% yield. Hydrogenolysis with Pd/C gave phosphatidic acid 34 in excellent yield, column chromatography being obsolete. With this strategy, 32 was synthesized in 3-steps in 53% overall yield, and mixed di-acyl phosphatidic acid 34 was synthesized in 4 steps in 37% overall yield.

**Scheme 2:**
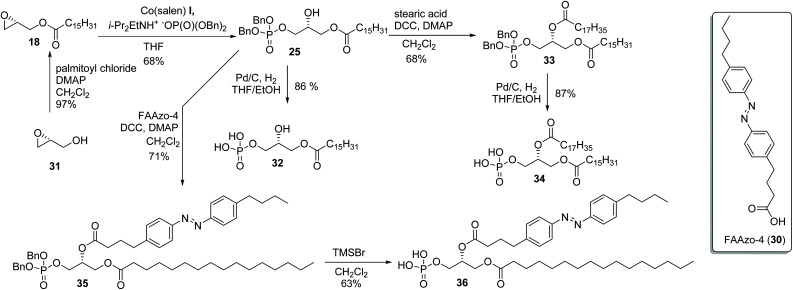
Synthesis of mono- and di-acyl phosphatidic acids.

The yields obtained for these straightforward phosphatidic acids are similar to those observed by Konradsson^15^ and our previous work.^22^ The Co(iii)–salen catalyst, however, is a much milder Lewis acid than the BF_3_ used in these methods, and allows the presence of several sensitive functional groups. We indeed found that protecting groups (Table 1) such as TBDPS (27) and trityl (28) gave excellent yields in the ringopening reaction, even though they introduce in addition significant steric bulk. We were also pleased to see that we could phosphorylate enantiopure epichlorohydrin (26) in a good yield of 61%, leaving the chloride untouched. Even the highly acid-sensitive geranylgeranyl substituted glycidol was a versatile substrate (24), which provides access to geranylgeranyl glycerol phosphate, an archaeal lipid.^31,32^

**Table 1:** Substituted glycidols in the phosphate ring-opening reaction^a^

Entry	Ring-opened product	Yield
1	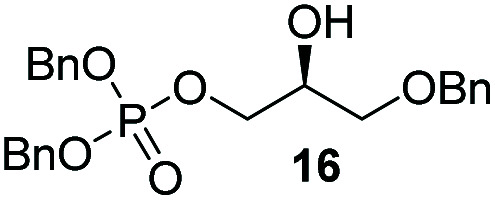	84%
2^b^	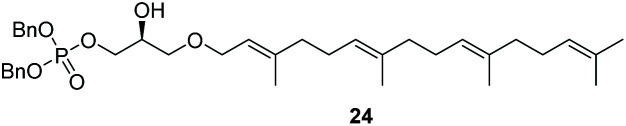	64%
3	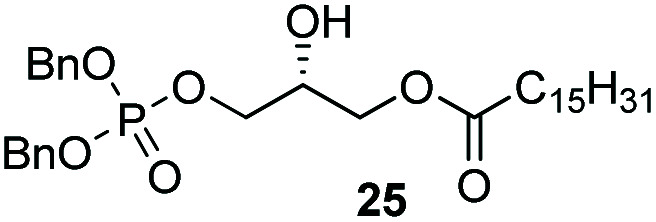	68%
4	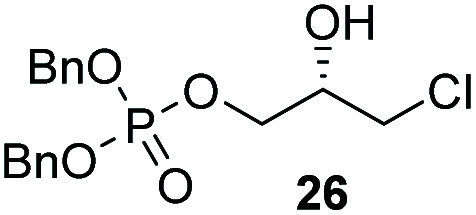	61%
5	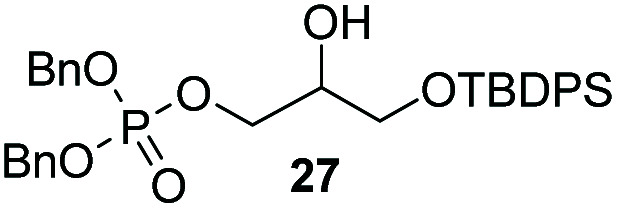	85%
6	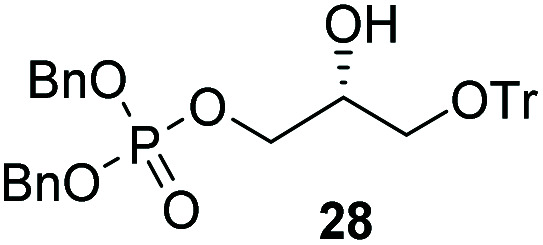	85%

a Optimal conditions: 10% of cat I, 1 equiv. of purified dibenzylphosphate dipea salt, 16 h reaction time, yield determined after purification by column chromatography.

b Dibenzylphosphate dipea salt was made *in situ*.

It turned out that the method is also very useful to prepare photoswitchable phosphatidic acids. Starting from 25, (Scheme 2) a 4-butyl-azo-4 : 0-acid-1 (FAAzo-4, 30) group, the most common photo-switchable lipophilic tail, was installed with a DCC-mediated coupling reaction. Subsequent deprotection of 35 with TMSBr provided the desired photoswitchable phosphatidic acid mimic 36, with the diazo switch intact, in 4-steps from (*R*)-glycidol (31). Similar switchable lipids have been prepared in more steps but a higher yield.^9^

In conclusion, dibenzyl phosphate can be used in an efficient Jacobsen-type epoxide ring-opening reaction. The reaction is compatible with a variety of substituents on the hydroxy group of glycidol. This finding is used for a “best-in-class” synthesis of mono and di-acyl phosphatidic acids. The strategy provides the lipids in a limited number of synthetic steps, in high yields and without acyl shift. As an application, a photo-switchable diacyl-phosphatidic acid has been prepared, demonstrating the utility of the method in chemical biology.

## Conflicts of interest

There are no conflicts to declare.

## Supplementary Material

OB-020-D2OB00168C-s001
