# Supplementary material for: Synthesis of phosphatidic acids via cobalt(salen) catalyzed epoxide ring-opening with dibenzyl phosphate
Source: Org Biomol Chem. 2022 Mar 3;20(11):2200–4. doi: 10.1039/d2ob00168c (PMC8924959; doi:10.1039/d2ob00168c)
Supplement: OB-020-D2OB00168C-s001 [file OB-020-D2OB00168C-s001.pdf]

Electronic Supplementary Material (ESI) for Organic & Biomolecular Chemistry.
This journal is © The Royal Society of Chemistry 2022

Supporting information

Synthesis of Phosphatidic Acids via Cobalt(salen) Catalyzed Epoxide Ring-opening with Dibenzyl Phosphate

Ruben L.H. Andringa, Marijn Jonker, Adriaan J. Minnaard*

Stratingh Institute for Chemistry, University of Groningen, Nijenborgh 7, 9747 AG, Groningen, The Netherlands.

*Correspondence should be adressed to: A.J.Minnaard@rug.nl

Contents

General Information S2
Screening tables S3
Experimental procedures S5
Spectral Data S11
References S30

General Information

All reactions were carried out under a nitrogen atmosphere using oven-dried glassware and using standard Schlenk techniques unless otherwise mentioned. Reaction temperature refers to the temperature of the oil bath. All reagents and catalysts were purchased from Sigma-Aldrich, Acros, J&K Scientific and TCI Europe and used without further purification unless otherwise mentioned. Any purification of reagents was performed following the methods described in; Armarego, W. L., & Chai, C. L. L. (2013) *Purification of laboratory chemicals. Butterworth-Heinemann*. TLC analysis was performed on Merck silica gel 60/Kieselguhr F254, 0.25 mm. Compounds were visualized using either Seebach's reagent (a mixture of phosphomolybdic acid (25 g), cerium (IV) sulfate (7.5 g), H_2O (500 mL) and H_2SO_4 (25 mL)), 2,4-DNP stain (2,4-dinitrophenylhydrazine (12 g), conc. sulfuric acid (60 ml), water (80 ml), ethanol (200 ml)), bromocresol green (a mixture of bromocresol green (0.04 g) in EtOH (100 mL), 0.1 M NaOH added until mixture turns blue), Phosphomolybdic acid (PMA) stain (a mixture of phosphomolybdic acid (10 g) in EtOH (100 mL)) or elemental iodine. Flash chromatography was performed using SiliCycle silica gel type SiliaFlash P60 (230 – 400 mesh).GC-MS measurements were performed with an HP 6890 series gas chromatography system equipped with an HP1 or HP5 column (Agilent Technologies, Palo Alto, CA), and equipped with an HP 5973 mass sensitive detector. High resolution mass spectra (HRMS) were recorded on a Varian AMX400 (400, 100.6 and 162 MHz, respectively) using $CDCl_3$ as solvent unless stated otherwise. Chemical shift values are reported in ppm with the solvent resonance as the internal standard ($CDCl_3$: δ 7.26 for ^1H, δ 77.16 for ^{13}C). Data are reported as follows: chemical shifts (δ), multiplicity (s = singlet, d = doublet, dd = double doublet, ddd = double double doublet, td = triple doublet, t = triplet, q = quartet, b = broad, m = multiplet), coupling constants J (Hz), and integration. Optical rotations were measured on a Schmidt+Haensch polarimeter (Polartronic MH8) with a 10 cm cell (c given in g/mL) at ambient temperature (±20 °C).

Screening tables

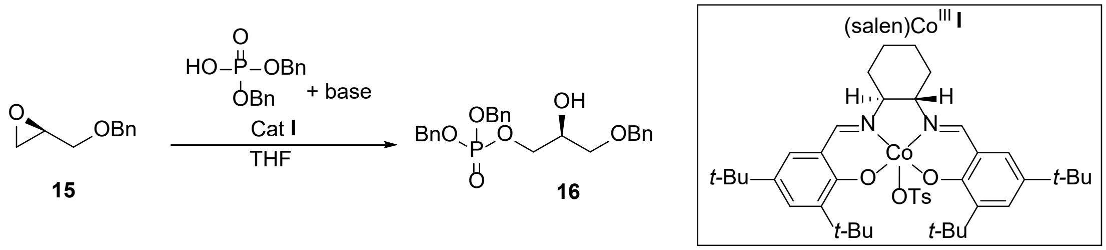

Table 1. Base screening with epoxide **15** and cat **I**[a]

Entry	base	Yield
**1**	Cs_2CO_3 (1.1 eq)	0%
**2**	2,6-lutidine (1.1 eq)	55%
**3**	2,6-lutidine (0.05 eq)	18%
**4**	2,6-di-tert-butylpyridine (1.1 eq)	15%
**5**	DIPEA (0.05 eq)	53%
**6**	DIPEA (0.05 eq)[b]	26%
**7**	DIPEA (1.1 eq)	78%
**8**	DIPEA[c] (1 eq)	84%

[a] *conditions:* 10% of cat **I**, 1 equiv of dibenzylphosphoric acid, 16 h reaction time, yield determined after purification by column chromatography [b]2 equiv of dibenzylphosphoric acid was used, [c] a 1:1 mixture of DIPEA and dibenzylphosphoric acid was stirred in CH_2Cl_2, the resulting Hünig's salt was purified (silica column, 5% MeOH in EtOAc), and then used in the reaction.

Scheme 1: Ring-opening phosphorylation with cat **II**

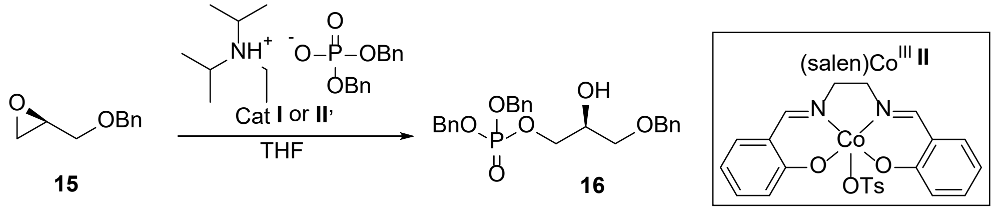

Table 2. Catalyst screening for the ring opening of epoxide **15**

Entry	Catalyst	Phosphate	Yield
**1**	**I** (5%)	Hünig's salt	70%
**2**	**II** (5%)	Hünig's salt	55%
**3**	**I** (7.5%)	Hünig's salt	80%
**4**	**II** (7.5%)	Hünig's salt	62%
**5**	**I** (10%)	Hünig's salt	84%
**6**	**II** (10%)	Hünig's salt	65%

Table 3. Base screening for the ring opening of epoxide **15**

Entry	Base	Yield	Remarks
**1**	Cs_2CO_3 (1.1 eq)	0%	-
**2**	2,6-Lutidine (1 eq)	55%	-
**3**	2,6-Lutidine (0.05 eq)	18%	-
**4**	2,6-di-tert-butylpyridine (1 eq)	15%	-
**5**	DIPEA (0.05 eq)	53%	-
**6**	DIPEA (0.05 eq)	26%	2 eq of phosphate was used
**2**	DIPEA (1 eq)	45%	2 eq of epoxide was used

Experimental procedures

General procedure for the epoxide ring-opening with a Co-salen catalyst and dibenzyl phosphate as the nucleophile:

To a vial containing a stirrer egg was added dibenzylphosphate DIPEA salt[i] (1.54 g, 4.3 mmol, 1 equiv) and the oxirane (4.26 mmol, 1 equiv) in dry THF (2 mL) under N_2 atmosphere. Co-salen catalyst **I** (0.426 mmol, 10 mol%) was added and the green mixture was allowed to stir for 16 h under an oxygen atmosphere. The viscous mixture was purified by column chromatography (50% EtOAc in pentane) which yielded the desired phosphate as a viscous oil.[ii]

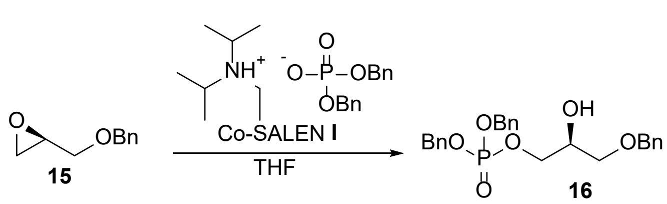

(*S*)-Dibenzyl (3-(benzyloxy)-2-hydroxypropyl) phosphate **16** was synthesized via the general procedure starting from epoxide **15** (1.6 g, 3.61 mmol, 84%).

Spectral data matched with those previously reported of the racemic compound.[1]

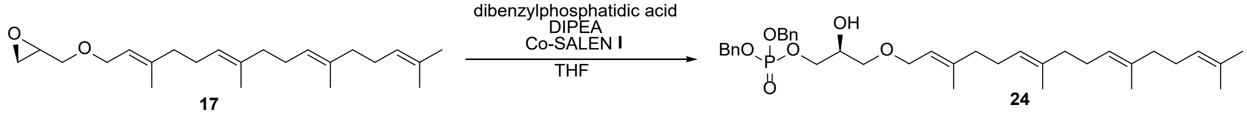

(*S*)-Dibenzyl (2-hydroxy-3-(geranylgeranyl-oxy)propyl) phosphate **24** was synthesized via the general procedure starting from epoxide **17** (60 mg 0.1 mmol, 64%).

^{1}H NMR (400 MHz, Chloroform-*d*) δ 7.34 (s, 10H), 5.31 (t, *J* = 6.9 Hz, 1H), 5.17 – 5.01 (m, 7H), 4.14 – 4.01 (m, 2H), 3.99 (d, *J* = 6.8 Hz, 2H), 3.93 (p, *J* = 5.6 Hz, 1H), 3.42 (d, *J* = 5.4 Hz, 2H), 2.99 (s, 1H), 2.17 – 1.92 (m, 12H), 1.68 (s, 3H), 1.65 (s, 3H), 1.60 (s, 9H).
^{13}C NMR (101 MHz, cdcl$_3$) δ 140.6, 135.8, 135.7, 135.4, 135.0, 131.3, 128.7, 128.1, 124.5, 124.3, 123.9, 120.4, 70.0, 69.6, 69.6, 69.5, 69.5, 69.5, 69.4, 69.2, 69.2, 67.9, 39.8, 39.8, 39.7, 26.8, 26.7, 26.4, 25.8, 17.8, 16.6, 16.1, 16.1.
^{31}P NMR (162 MHz, Chloroform-*d*) δ -0.15.
$[\alpha]_D^{20}$= +2.0 (c = 1 in $CHCl_3$).
HRMS-ESI$^+$ (*m/z*): $[M]^+$ calculated for $C_{37}H_{53}PO_6Na^+$, 647.347; found, 647.345

[i] The ammonium salt was made by addition of 1 equiv of dibenzylphosphoric acid with 1 equiv of DIPEA in a minimal amount of CH_2Cl_2, after stirring for 5 min the mixture was purified by flash column chromatography (EtOAc/MeOH 20:1). *In situ* generation of the salt is also possible, by stirring 5 minutes in dry THF.
[ii] Optical rotations of the phosphorylated products were not reliable as a minute amount of catalyst co-eluted with the products, this also resulted in green/brown coloured products. In subsequent steps the trace amounts could be separated out effortlessly.

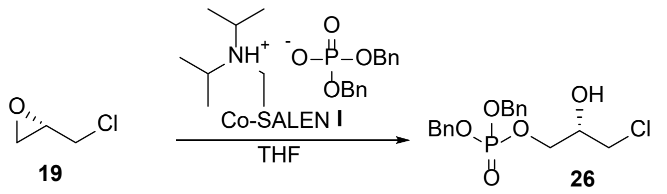

(*S*)-Dibenzyl (3-chloro-2-hydroxypropyl) phosphate **25** was synthesized via the general procedure starting from epoxide **19** (270 mg, 0.73 mmol, 61%).

^{1}H NMR (400 MHz, $CDCl_3$) δ 7.34 (d, *J* = 2.9 Hz, 10H), 5.14 – 4.97 (m, 4H), 4.16 – 4.02 (m, 2H), 3.94 (p, *J* = 5.3 Hz, 1H), 3.50 (d, *J* = 5.6 Hz, 2H), 2.03 (s, 1H).
^{13}C NMR (101 MHz, $CDCl_3$) δ 135.53, 135.47, 128.8, 128.7, 128.1, 128.1, 70.0, 69.9, 69.84, 69.81, 69.78, 69.76, 68.4, 68.3, 44.7.
HRMS-ESI+ (m/z): $[M]^+$ calculated for $C_{17}H_{20}O_5ClPNa$, 393.063; found, 393.063.

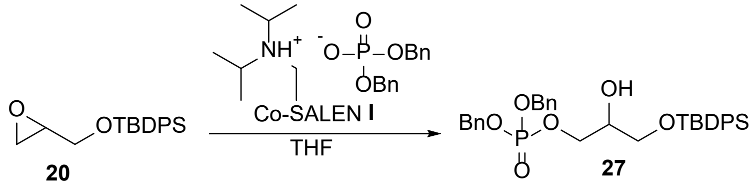

Dibenzyl (3-((tert-butyldiphenylsilyl)oxy)-2-hydroxypropyl) phosphate **27** was synthesized via the general procedure starting from epoxide **20** (350 mg, 0.59 mmol, 85%).

^{1}H NMR (400 MHz, $CDCl_3$) δ 7.71 – 7.48 (m, 4H), 7.49 – 7.29 (m, 16H), 5.10 – 4.99 (m, 4H), 4.21 – 4.05 (m, 2H), 3.89 (t, *J* = 5.5 Hz, 1H), 3.73 – 3.61 (m, 2H), 2.76 (s-br, 1H), 1.06 (s, 9H).
^{13}C NMR (101 MHz, $CDCl_3$) δ 135.7, 135.5, 135.5, 132.9, 129.9, 128.61, 128.59, 128.0, 127.8, 77.3, 70.6, 70.6, 69.6, 69.5, 68.8, 68.8, 63.9, 26.8, 19.2.
^{31}P NMR (162 MHz, $CDCl_3$) δ 0.03.
HRMS-ESI+ (m/z): $[M]^+$ calculated for $C_{33}H_{39}O_6PSiNa$, 613.215; found, 613.216.

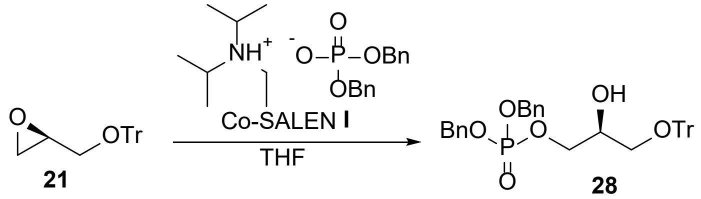

(*S*)-Dibenzyl (2-hydroxy-3-(trityloxy)propyl) phosphate **21** was synthesized via the general procedure starting from epoxide **28** (270 mg, 0.45 mmol, 85%).

^{1}H NMR (400 MHz, $CDCl_3$) δ 7.63 – 7.10 (m, 25H), 5.09 – 4.95 (m, 4H), 4.18 – 4.01 (m, 2H), 3.96 – 3.85 (m, 1H), 3.26 – 3.10 (m, 2H).
^{13}C NMR (101 MHz, $CDCl_3$) δ 143.6, 128.6, 128.0, 127.9, 127.1, 86.8, 69.8, 69.8, 69.6, 69.5, 69.5, 63.7.
^{31}P NMR (162 MHz, $CDCl_3$) δ -0.03.
HRMS-ESI+ (m/z): $[M]^+$ calculated for $C_{36}H_{35}O_6PNa$, 617.207; found, 617.207.

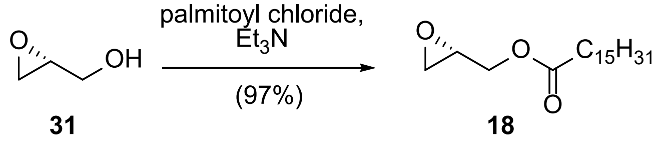

To a cooled (0 °C, ice/water bath) solution of (R)-oxiran-2-ylmethanol (2 g, 27.0 mmol), and triethylamine (4.5 mL, 32 mmol, 1.2 equiv) in THF (90 mL) was added palmitoyl chloride (9.8 mL, 32.4 mmol, 1.2 equiv) in a drop-wise fashion. The mixture was stirred for 16 h, during which time it was allowed to warm up to rt. Water was added and the mixture was transferred to a separatory funnel and was subsequently extracted with EtOAc (3x), the organic layers were combined, dried over $MgSO_4$, filtered and concentrated *in vacuo*. The crude was further purified by flash column chromatography (30% ether in pentane) which afforded the desired epoxide **18** in 97% yield as a white solid.

^{1}H NMR (400 MHz, $CDCl_3$) δ 4.40 (dd, *J* = 12.3, 3.1 Hz, 1H), 3.91 (dd, *J* = 12.3, 6.3 Hz, 1H), 3.20 (ddt, *J* = 6.1, 4.1, 2.9 Hz, 1H), 2.84 (dd, *J* = 4.9, 4.1 Hz, 1H), 2.64 (dd, *J* = 4.9, 2.6 Hz, 1H), 2.34 (t, *J* = 7.6 Hz, 2H), 1.63 (t, *J* = 7.3 Hz, 2H), 1.25 (s, 16H), 0.87 (t, *J* = 6.8 Hz, 3H).
^{13}C NMR (101 MHz, $CDCl_3$) δ 173.5, 64.7, 49.4, 44.6, 34.1, 31.9, 29.67, 29.66, 29.65, 29.63, 29.62, 29.57, 29.4, 29.3, 29.2, 29.1, 24.9, 22.7, 14.1.
$[\alpha]_D^{20}$= -13.3 (c = 1 in $CHCl_3$).
HRMS-ESI+ (m/z): $[M]^+$ calculated for $C_{19}H_{37}O_3$, 313.266; found, 313.266.

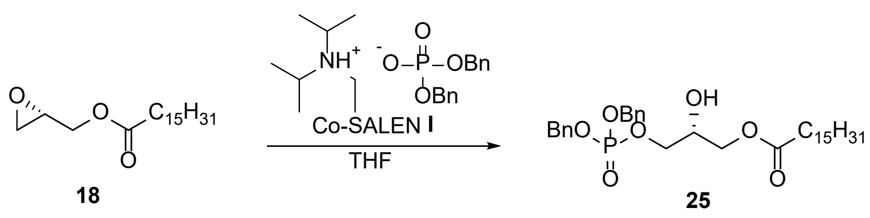

(R)-3-((Bis(benzyloxy)phosphoryl)oxy)-2-hydroxypropyl palmitate **25** was synthesized via the general procedure starting from epoxide **18** (250 mg, 0.43 mmol, 68%).[iii]

^{1}H NMR (400 MHz, $CDCl_3$) δ 7.35 (d, *J* = 2.2 Hz, 10H), 5.12 – 4.98 (m, 4H), 4.23 – 3.89 (m, 5H), 2.39 – 2.20 (m, 2H), 2.04 (s, 1H), 1.75 – 1.51 (m, 2H), 1.36 – 1.17 (m, 24H), 0.88 (t, *J* = 6.7 Hz, 3H).
^{13}C NMR (101 MHz, $CDCl_3$) δ 173.9, 135.7, 128.9, 128.8, 128.79, 128.77, 128.2, 69.88, 69.85, 68.92, 68.85, 64.4, 34.2, 32.1, 29.8, 29.8, 29.8, 29.74, 29.73, 29.60, 29.58, 29.5, 29.4, 29.3, 25.0, 22.8, 14.3.
^{31}P NMR (162 MHz, $CDCl_3$) δ 0.05.
HRMS-ESI+ (m/z): $[M]^+$ calculated for $C_{33}H_{51}O_7PNa$, 613.327; found, 613.327.

[iii] Compound was isolated with a small amount of co-eluting impurities which could be removed in the subsequent step.

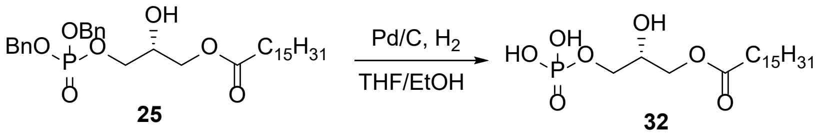

Dibenzyl phosphate **25** (110 mg, 0.19 mmol) was dissolved in a 1:1 mixture of THF/EtOH (3.8 mL). The mixture was degassed via freeze pump thaw technique (3x) before $Pd(OH)_2$ on carbon (53 mg, 0.04 mmol, 20 mol%, 10% Pd) was added. Hydrogen atmosphere was established and the mixture was allowed to stir vigorously for 16 h before it was filtered over celite, the filtrate was concentrated *in vacuo* which yielded the desired free phosphatidic acid **32** (58 mg, 0.17 mmol, 86%) as a pale-yellow solid.

^{1}H NMR (600 MHz, $CDCl_3/CD_3OD$ 4:1) δ 4.02 (s, 2H), 3.88 (s, 2H), 3.11 – 2.94 (m, 6H), 2.22 (m, 2H), 1.57 – 1.45 (m, 2H), 1.37 – 1.22 (m, 12H), 1.21 – 1.04 (m, 16H), 0.77 (t, *J* = 6.8 Hz, 3H).
^{13}C-45DEPT NMR (151 MHz, $CDCl_3$+MeOD) δ 77.4, 70.3, 64.91, 45.90, 34.1, 31.9, 29.62, 29.59, 29.58, 29.56, 29.4, 29.3, 29.2, 29.1, 24.8, 22.6, 13.4, 8.3.[iv]
^{31}P NMR (243 MHz, $CDCl_3/CD_3OD$ 4:1) δ -1.16
HRMS-ESI+ (m/z): $[M]^+$ calculated for $C_{19}H_{39}O_7PNa$, 433.233; found, 433.233.

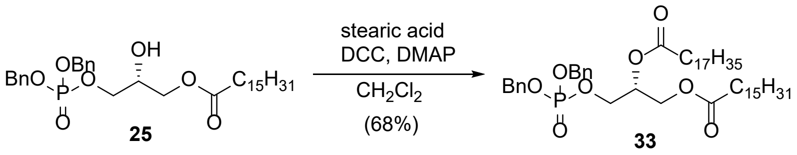

To a Schlenk flask equipped with stirrer egg was added dibenzyl phosphate **25** (90 mg, 0.16 mmol), stearic acid (53 mg, 0.19 mmol, 1.2 equiv) and DMAP (1 mg, 0.01 mmol, 5 mol%). The resulting mixture was cooled to 0 ºC (ice/water bath) and DCC (39 mg, 0.19 mmol, 1.2 equiv) was added, the resulting mixture was stirred for 16 h during which time it was allowed to warm up to rt. All volatiles were evaporated and the crude was further purified by flash chromatography (10% ether in pentane) which yielded the desired mixed di-acylglycerol **33** (90 mg, 0.11 mmol) in 68% yield as a white solid.

^{1}H NMR (400 MHz, $CDCl_3$) δ 7.34 (d, *J* = 2.1 Hz, 10H), 5.15 (p, *J* = 5.0 Hz, 1H), 5.10 – 4.97 (m, 4H), 4.34 – 3.94 (m, 4H), 2.36 – 2.20 (m, 4H), 1.57 (dtq, *J* = 11.7, 7.5, 4.3 Hz, 4H), 1.24 (d, *J* = 4.5 Hz, 52H), 0.87 (t, *J* = 6.7 Hz, 6H).
^{13}C NMR (101 MHz, $CDCl_3$) δ 173.2, 172.8, 135.62, 135.55, 128.63, 128.60, 128.58, 127.96, 127.94, 127.9, 69.53, 69.47, 69.4, 69.33, 69.25, 65.8, 65.40, 65.35, 61.6, 34.1, 34.0, 31.9, 29.69, 29.65, 29.62, 29.60, 29.47, 29.46, 29.35, 29.27, 29.11, 29.06, 24.8, 24.8, 22.7, 14.1.
^{31}P NMR (162 MHz, $CDCl_3$) δ -1.06, -1.66.
$[\alpha]_D^{20}$= -1.5 (c = 1 in $CHCl_3$).
HRMS-ESI+ (m/z): $[M]^+$ calculated for $C_{51}H_{85}O_8P_1Na$, 879.587; found, 879.588.

[iv] Due to poor solubility a DEPT-45 spectrum was obtained, this gives increased resolution but does not show signals of quaternary carbons.

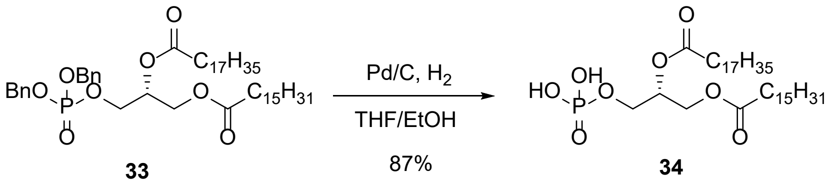

Mixed diacylglycerol phosphate **34** was prepared with the same synthetic procedure that was used for the synthesis of **32** (87% yield)

^{1}H NMR (600 MHz, $CDCl_3$+MeOD) δ 4.36 (dd, *J* = 12.0, 3.5 Hz, 2H), 4.17 (dd, *J* = 12.0, 6.4 Hz, 1H), 4.08 (t, *J* = 5.9 Hz, 2H), 3.33 (p, *J* = 1.6 Hz, 9H), 2.35 – 2.27 (m, 4H), 1.62 – 1.54 (m, 4H), 1.24 (s, 48H), 0.86 (t, *J* = 7.0 Hz, 6H).
^{13}C-45DEPT NMR (151 MHz, $CDCl_3$+MeOD) δ 77.49, 69.8, 62.1, 46.4, 34.1, 34.0, 31.8, 29.60, 29.56, 29.4, 29.3, 29.2, 29.0, 24.8, 22.6, 13.8, 8.5.[v]
^{31}P NMR (243 MHz, $CDCl_3$+MeOD) δ -0.24.
HRMS-ESI+ (m/z): $[M]^+$ calculated for $C_{37}H_{72}O_8P_1Na$, 699.494; found, 699.494.

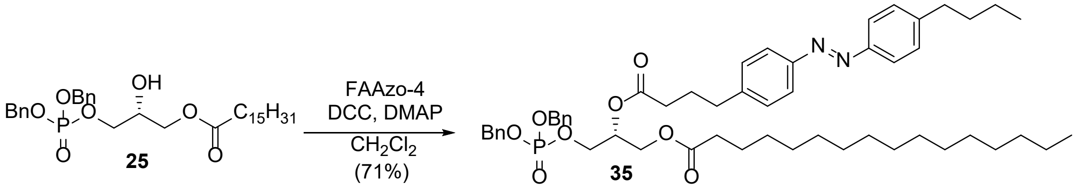

Photoswitchable mixed diacyl glycerol dibenzylphosphate **35** was prepared with the same synthetic procedure that was used for the synthesis of **33** (71%).

^{1}H NMR (400 MHz, $CDCl_3$) δ 7.91 – 7.75 (m, 4H), 7.39 – 7.23 (m, 14H), 5.18 (p, *J* = 5.1 Hz, 1H), 5.10 – 4.94 (m, 4H), 4.77 (s, 1H), 4.35 – 4.22 (m, 1H), 4.22 – 4.02 (m, 3H), 2.69 (t, *J* = 7.8 Hz, 4H), 2.38 – 2.18 (m, 4H), 2.02 – 1.90 (m, 2H), 1.84 (s, 2H), 1.70 – 1.61 (m, 2H), 1.60 – 1.49 (m, 2H), 1.39 (h, *J* = 7.3 Hz, 2H), 1.33 – 1.18 (m, 25H), 0.95 (t, *J* = 7.4 Hz, 3H), 0.88 (t, *J* = 6.8 Hz, 3H).
^{13}C NMR (101 MHz, $CDCl_3$) δ 173.3, 172.8, 172.5, 151.4, 151.1, 146.43, 146.42, 144.4, 135.70, 135.69, 135.6, 129.22, 129.17, 128.77, 128.73, 128.71, 128.08, 128.07, 127.97, 123.0, 122.9, 77.4, 69.72, 69.67, 69.64, 69.62, 69.55, 65.52, 65.46, 61.7, 35.7, 34.9, 34.1, 34.0, 33.6, 33.4, 33.3, 32.0, 29.80, 29.79, 29.8, 29.72, 29.58, 29.57, 29.5, 29.4, 29.2, 26.3, 26.2, 24.93, 24.86, 22.8, 22.4, 14.2, 14.0.
^{31}P NMR (162 MHz, $CDCl_3$) δ -0.99, -1.61.
$[\alpha]_D^{20}$= -4.5 (c = 1 in $CHCl_3$).
HRMS-ESI+ (m/z): $[M]^+$ calculated for $C_{53}H_{74}N_2O_8P_1Na$, 897.518; found, 897.519.

[v] Due to poor solubility a DEPT-45 spectrum was obtained, this gives increased resolution but does not show signals of quaternary carbons.

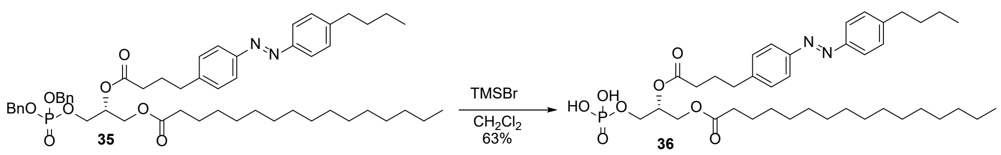

Dibenzylphosphate **35** (40 mg, 0.045 mmol) was dissolved in dry CH_2Cl_2 (0.9 mL) under N_2 atmosphere. The resulting solution was cooled to 0 ºC (ice/water bath) before TMSBr (13 µL, 0.1 mmol, 2.2 equiv) was added in a dropwise fashion. The resulting mixture was stirred for 2 h before water (0.1 mL) was added, subsequently all volatiles were evaporated *in vacuo*. The crude oil was purified by flash column chromatography ($CHCl_3$/MeOH/H_2O 80%/17%/3%) to afford the desired free photoswitchable mixed diacyl phosphatidic acid **36** (20 mg, 0.028 mmol, 63%) as an orange film.

^{1}H NMR (600 MHz, $CDCl_3$/CD_3OD 4:1) δ 7.75 – 7.55 (m, 3H), 7.24 – 7.02 (m, 4H), 6.92 (s, 0H), 6.69 – 6.52 (m, 0H), 5.13 (s, 1H), 4.49 – 4.13 (m, 2H), 3.01 – 2.87 (m, 3H), 2.66 – 2.43 (m, 3H), 2.33 – 1.98 (m, 3H), 1.89 – 1.68 (m, 2H), 1.57 – 1.28 (m, 4H), 1.29 – 0.88 (m, 31H), 0.85 – 0.61 (m, 6H).
^{13}C-45DEPT NMR (151 MHz, $CDCl_3$/CD_3OD 4:1) δ 133.04, 133.00, 132.96, 132.6, 132.2, 131.2, 126.7, 126.6, 124.7, 81.4, 71.3, 66.4, 39.4, 38.7, 38.0, 37.3, 35.8, 33.6, 33.5, 33.2, 33.1, 30.2, 28.7, 26.5, 26.2, 17.88, 17.86, 17.7, 12.3.[vi]
^{31}P NMR (243 MHz, $CDCl_3$/CD_3OD 4:1) δ 3.63.
HRMS-ESI+ (m/z): $[M]^+$ calculated for $C_{39}H_{61}N_2O_8P_1Na$, 739.406; found, 739.406.

[vi] Due to poor solubility a DEPT-45 spectrum was obtained, this gives increased resolution but does not show signals of quaternary carbons.

Spectral Data

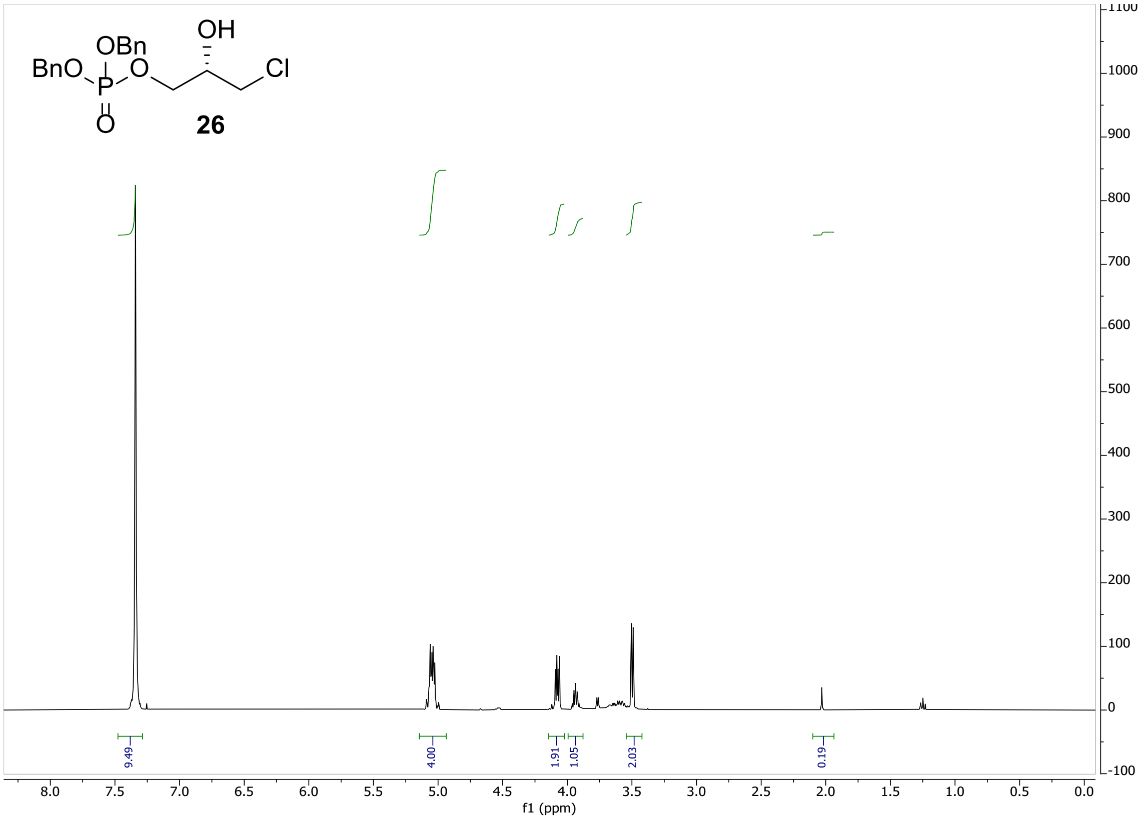

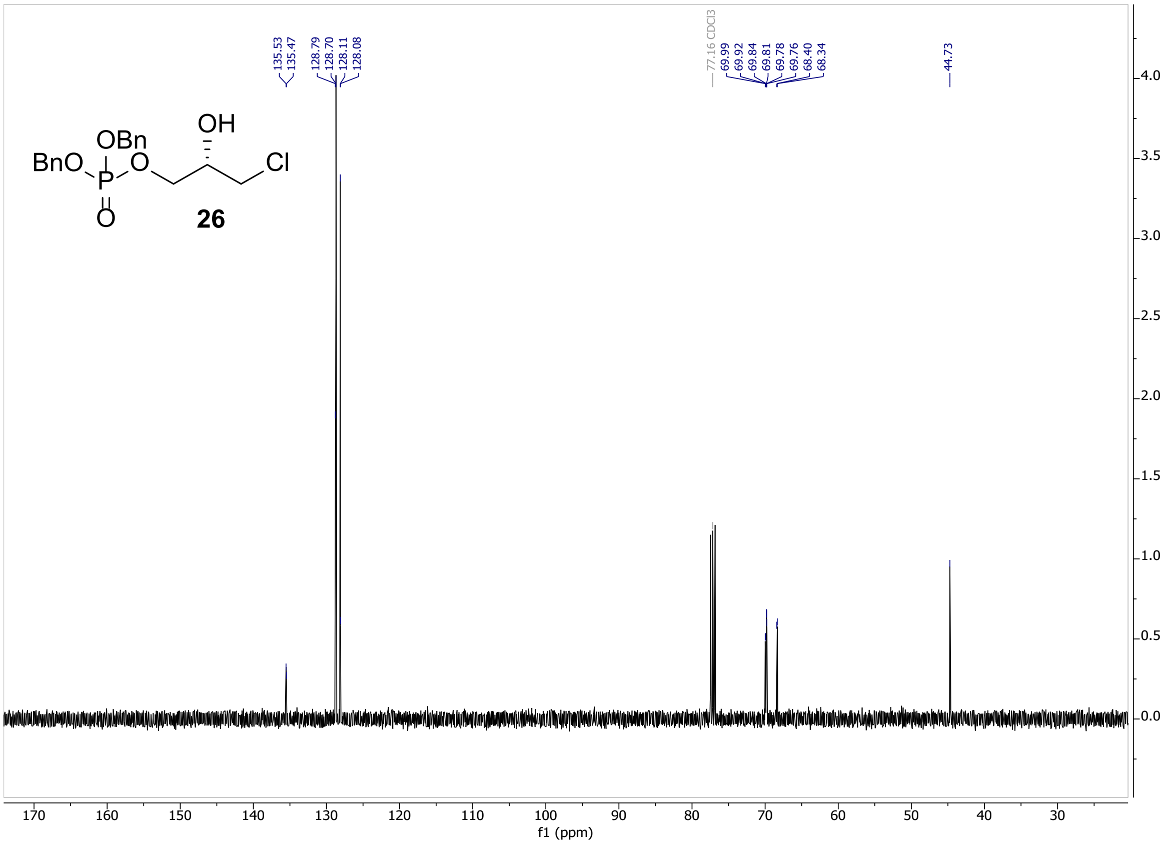

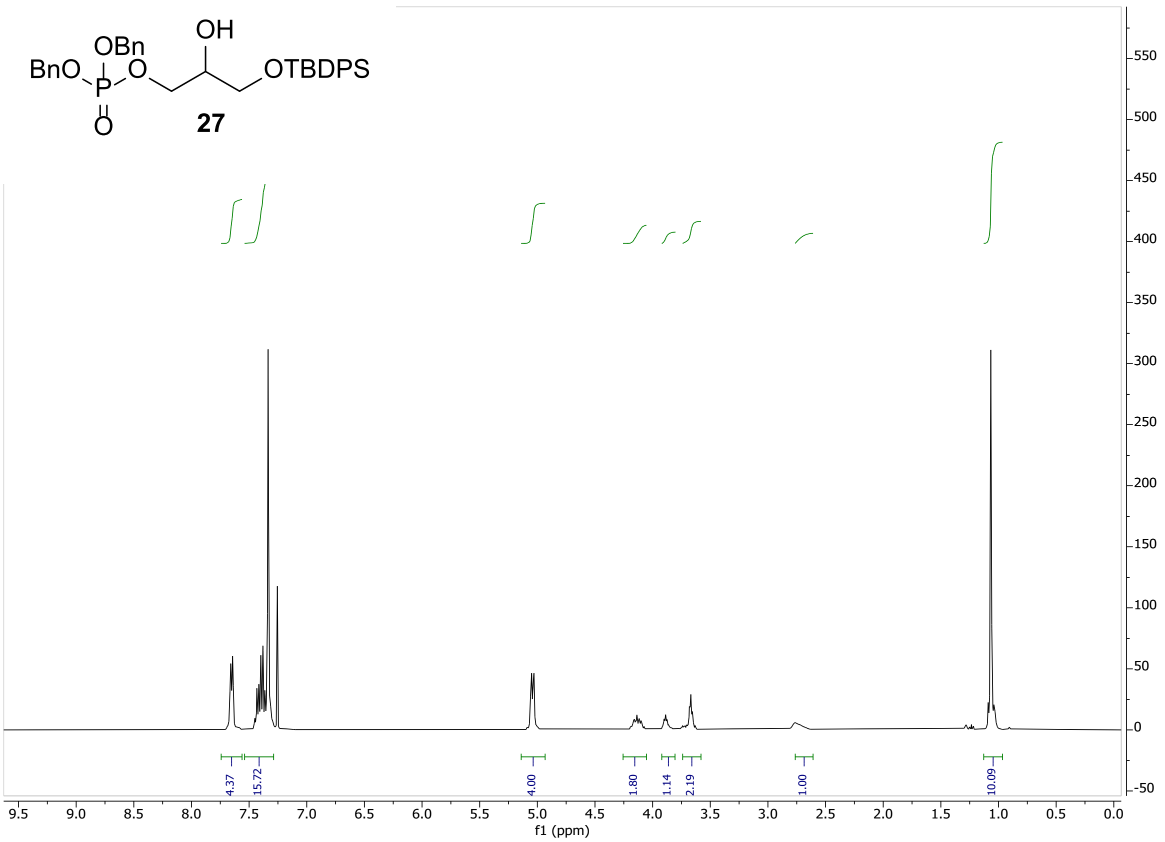
BnO
OBn
OH
OTBDPS
27
4.37
15.72
4.00
1.80
1.14
2.19
1.00
10.09
f1 (ppm)

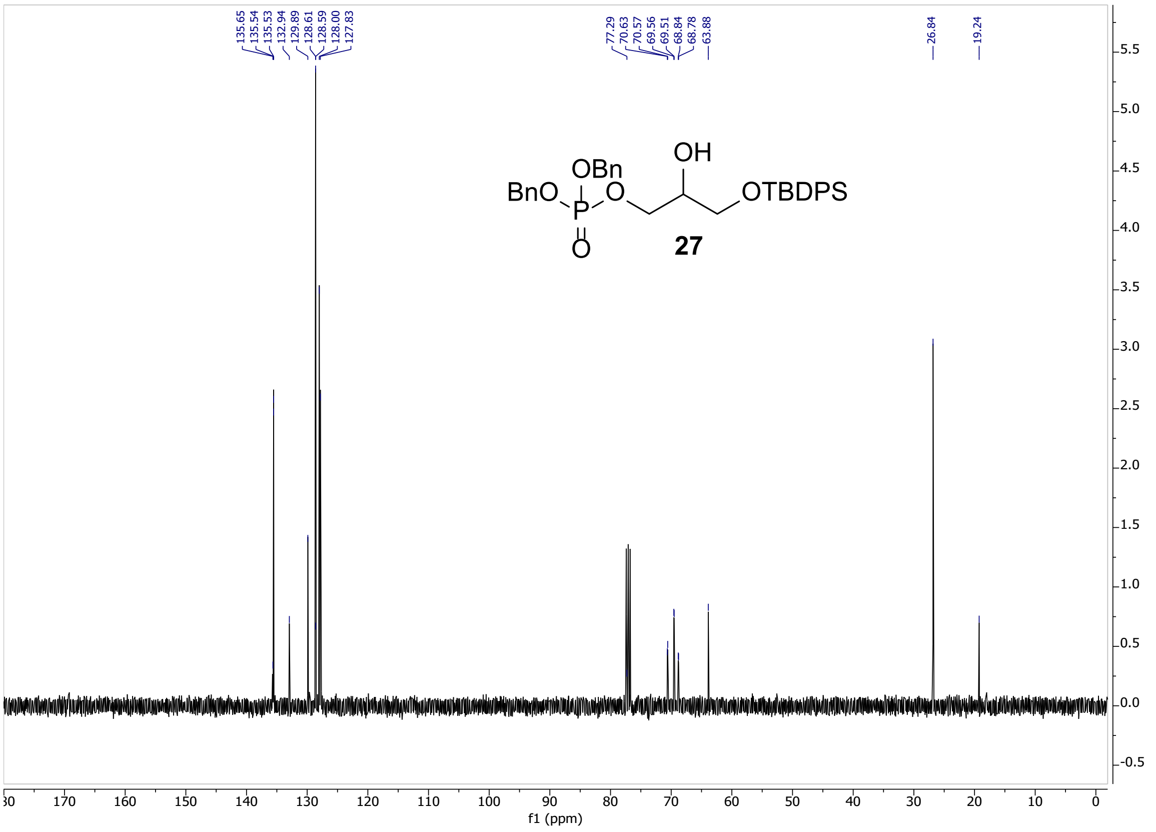
135.65
135.54
135.53
132.94
129.89
128.61
128.59
128.00
127.83
77.29
70.63
70.57
69.56
69.51
68.84
68.78
63.88
26.84
19.24
BnO
OBn
OH
OTBDPS
27
f1 (ppm)

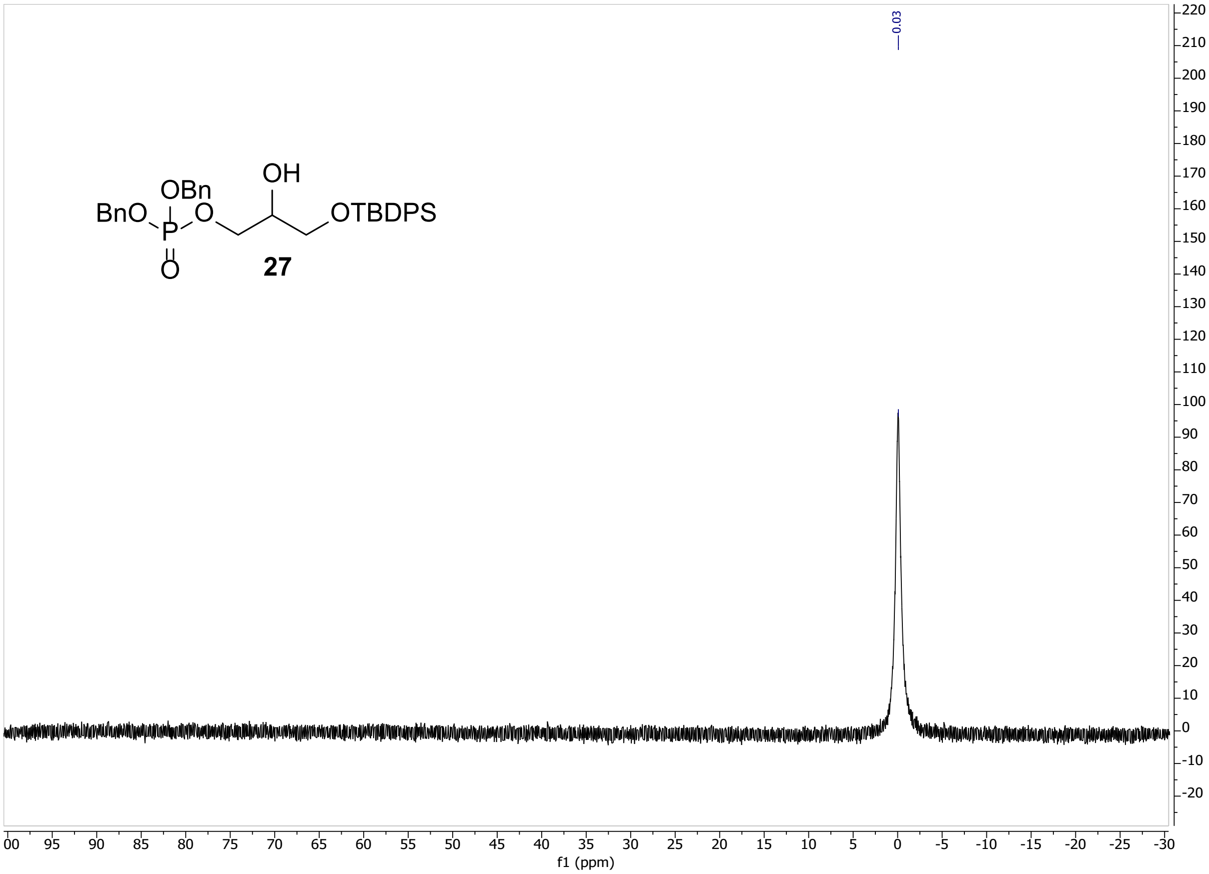

OBn
OH
BnO
P
O
OTBDPS
O
27
0.03
f1 (ppm)

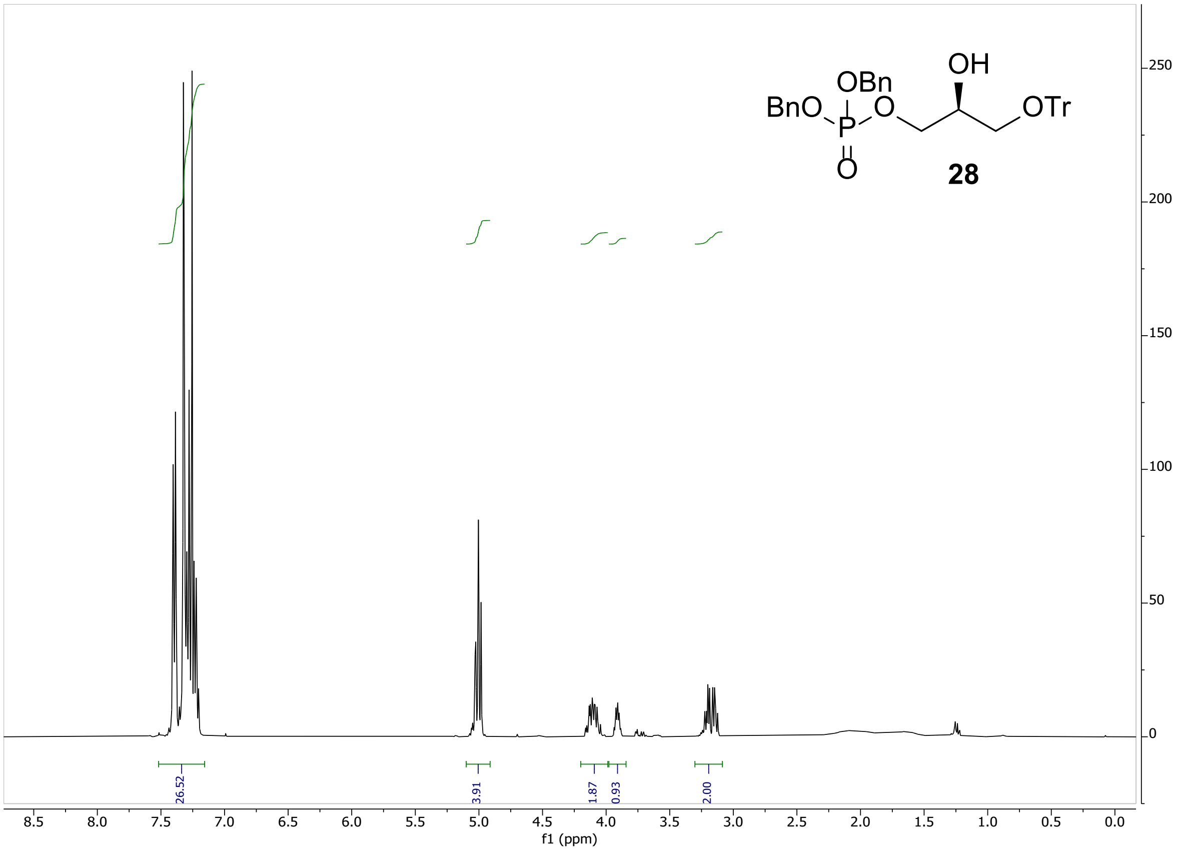

OBn
OH
BnO
P
O
OTr
O
28
26.52
3.91
1.87
0.93
2.00
f1 (ppm)

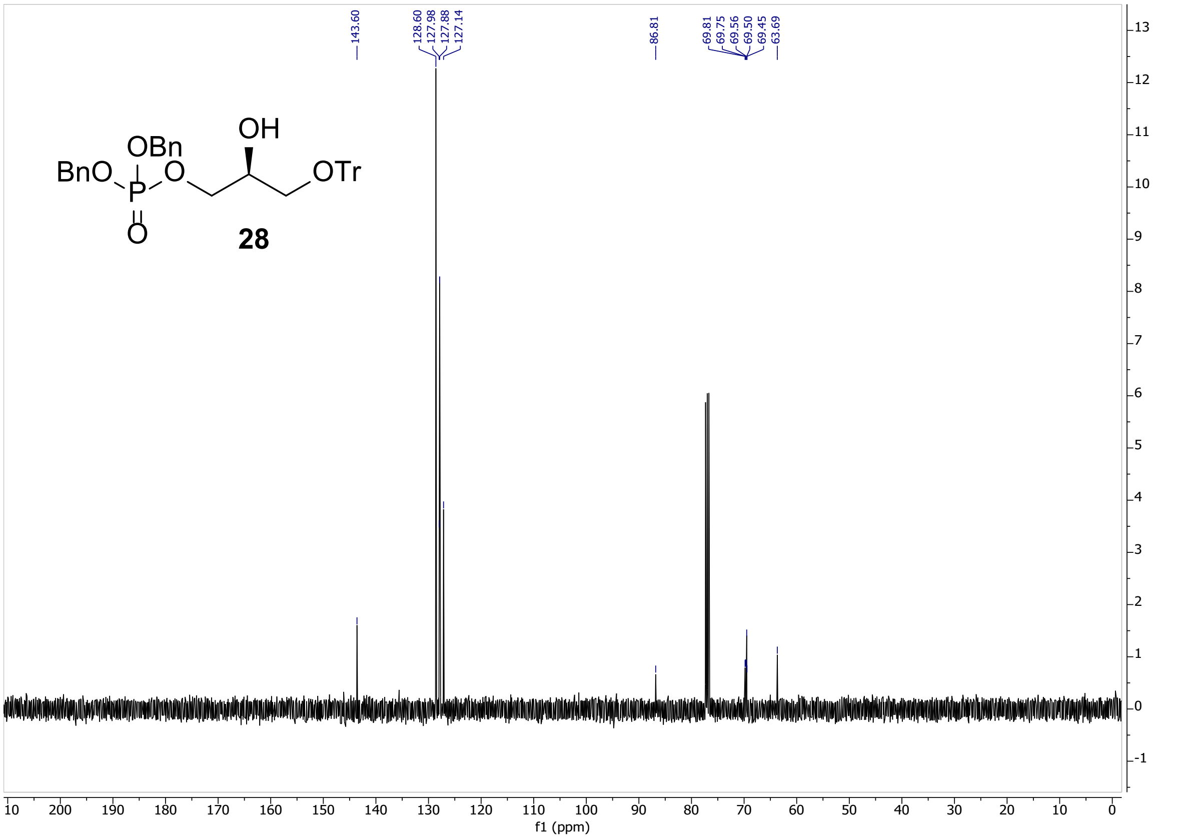

143.60
128.60
127.98
127.88
127.14
86.81
69.81
69.75
69.56
69.50
69.45
63.69
OH
OBn
BnO
P
O
OTr
O
28
f1 (ppm)

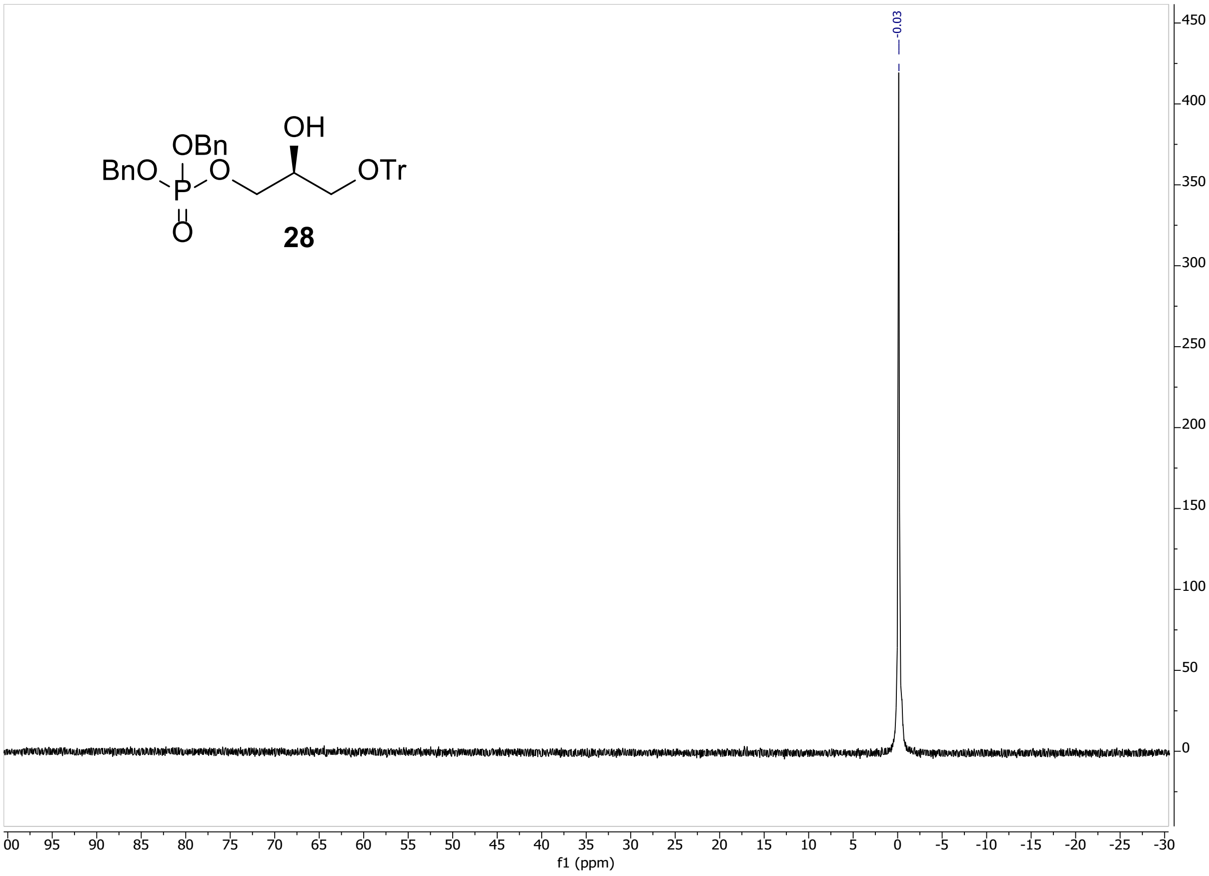
BnO
OBn
OH
O
OTr
28
-0.03
f1 (ppm)

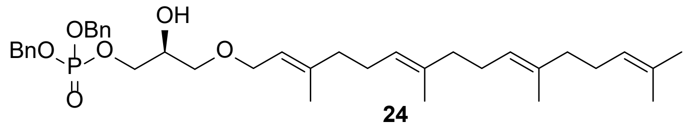
OBn
OH
BnO
P
O
O
O

24

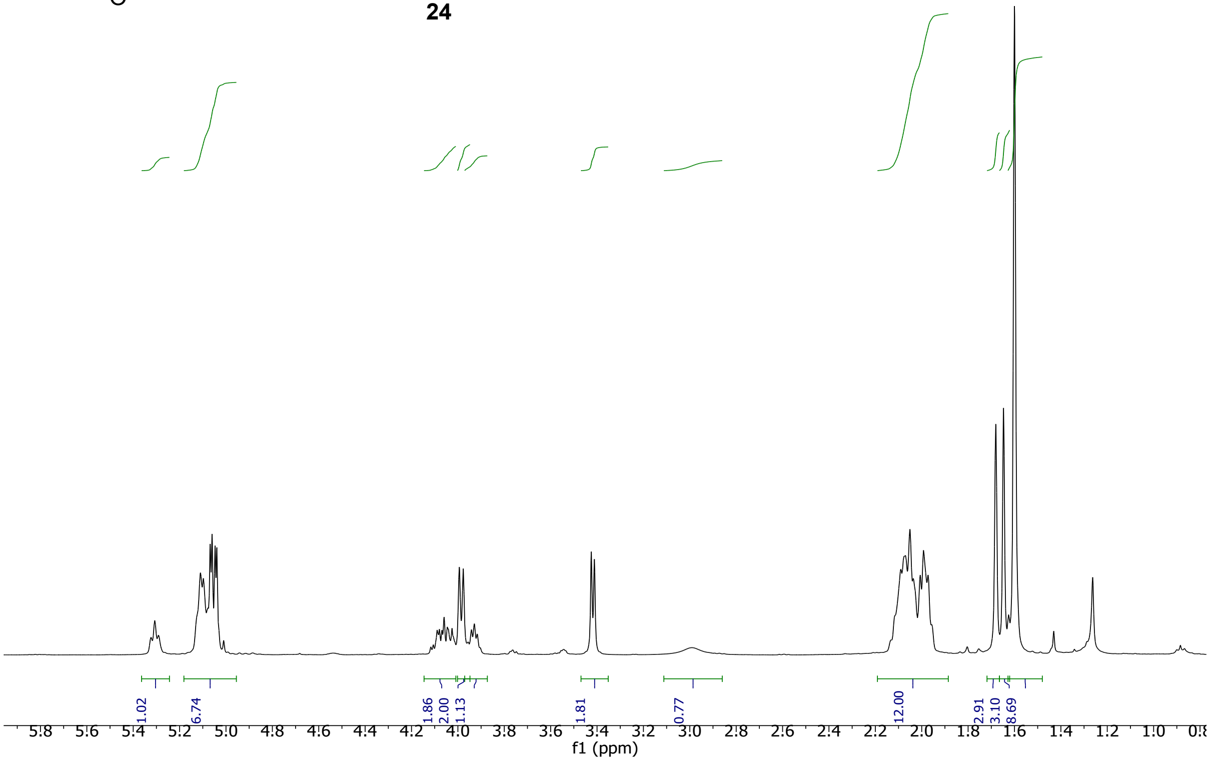
1.02
6.74
1.86
2.00
1.13
1.81
0.77
12.00
2.91
3.10
8.69
5.8 5.6 5.4 5.2 5.0 4.8 4.6 4.4 4.2 4.0 3.8 3.6 3.4 3.2 3.0 2.8 2.6 2.4 2.2 2.0 1.8 1.6 1.4 1.2 1.0 0.8
f1 (ppm)

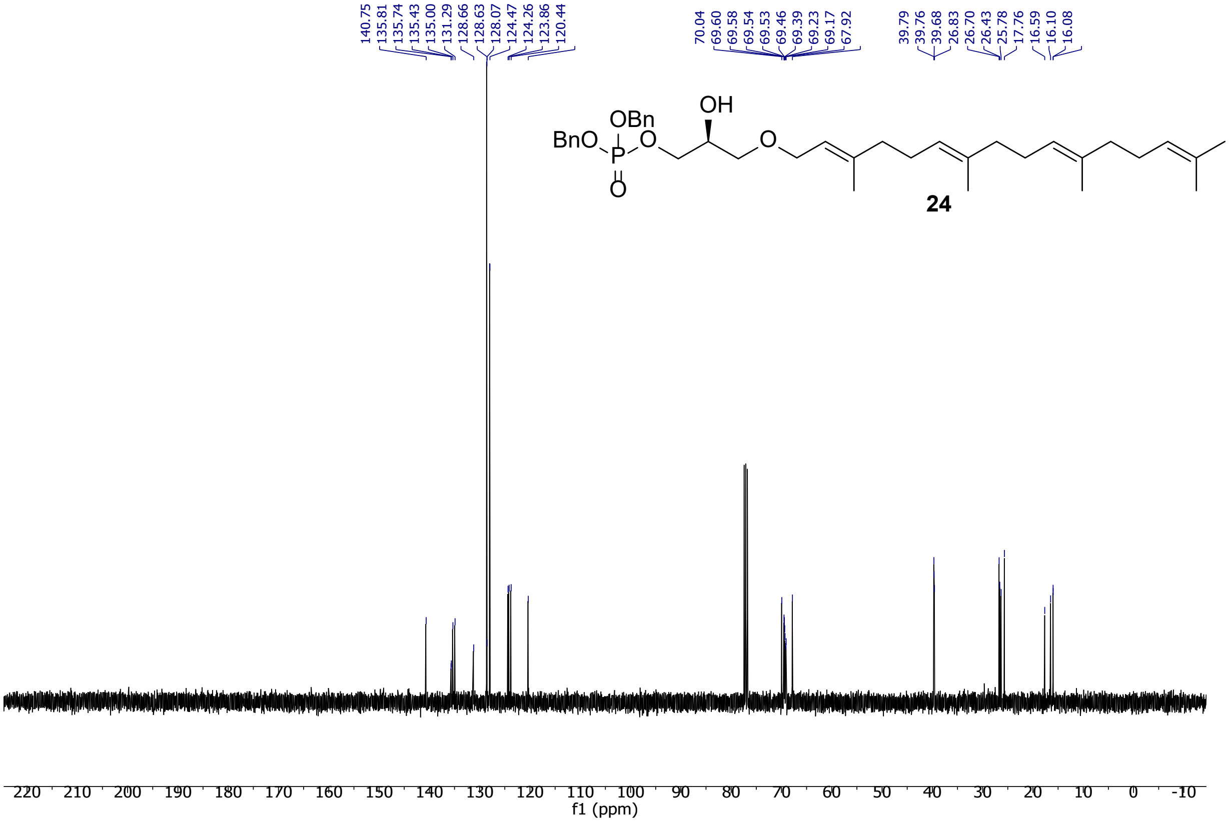
140.75
135.81
135.74
135.43
135.00
131.29
128.66
128.63
128.07
124.47
124.26
123.86
120.44
70.04
69.60
69.58
69.54
69.53
69.46
69.39
69.23
69.17
67.92
39.79
39.76
39.68
26.83
26.70
26.43
25.78
17.76
16.59
16.10
16.08
OBn
OH
BnO
P
O
O
O
24
220 210 200 190 180 170 160 150 140 130 120 110 100 90 80 70 60 50 40 30 20 10 0 -10
f1 (ppm)

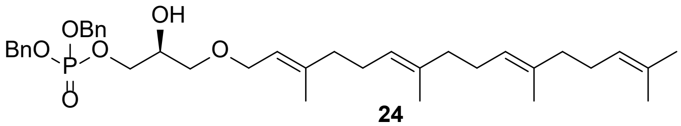
OBn
OH
BnO
P
O
O
O
O

24

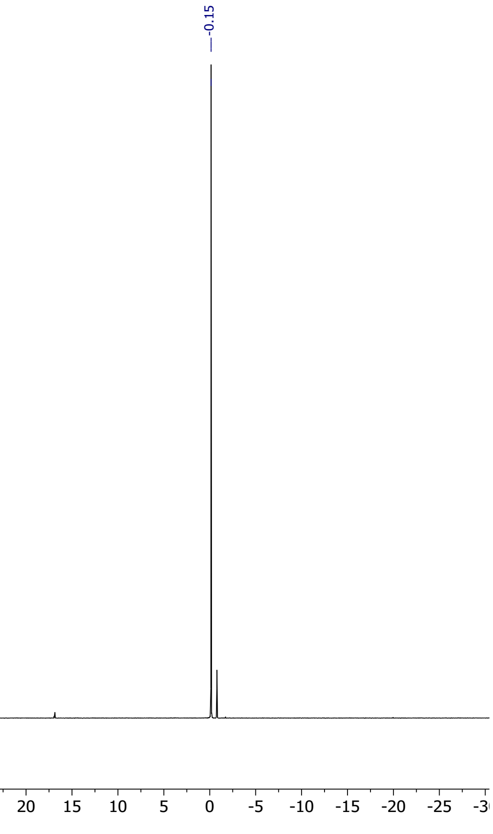
-0.15
00 95 90 85 80 75 70 65 60 55 50 45 40 35 30 25 20 15 10 5 0 -5 -10 -15 -20 -25 -3
f1 (ppm)

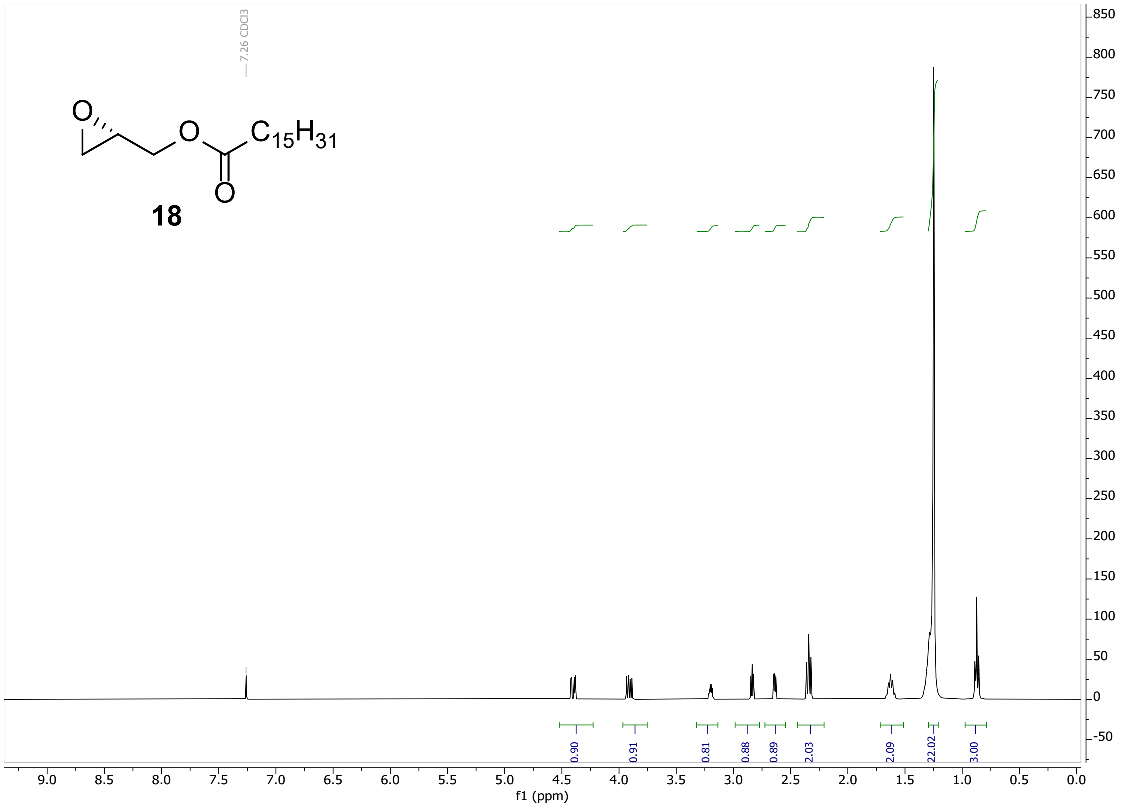
7.26 CDCl3
O
O
C15H31
O
18
0.90
0.91
0.81
0.88
0.89
2.03
2.09
22.02
3.00
f1 (ppm)

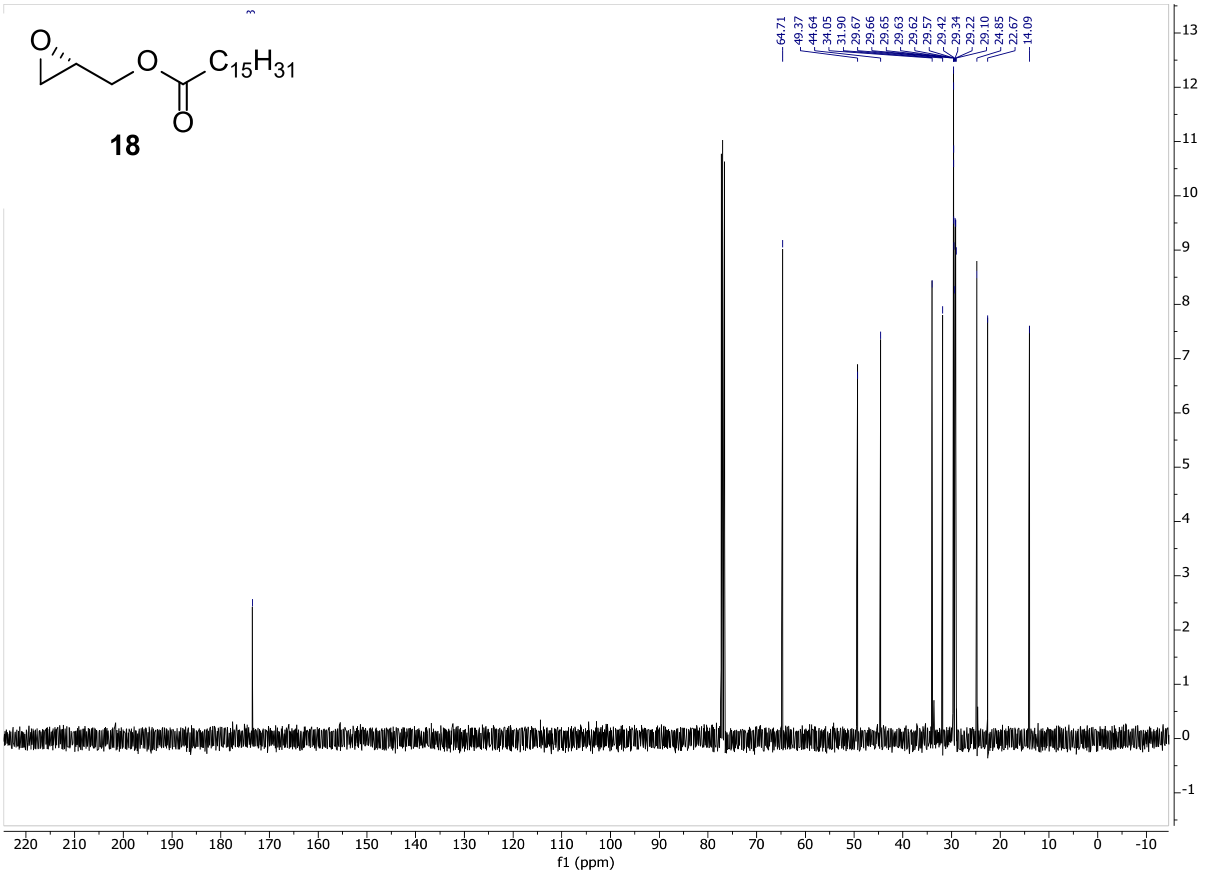
64.71
49.37
44.64
34.05
31.90
29.67
29.66
29.65
29.63
29.62
29.57
29.42
29.34
29.22
29.10
24.85
22.67
14.09
O
O
C15H31
O
18
f1 (ppm)

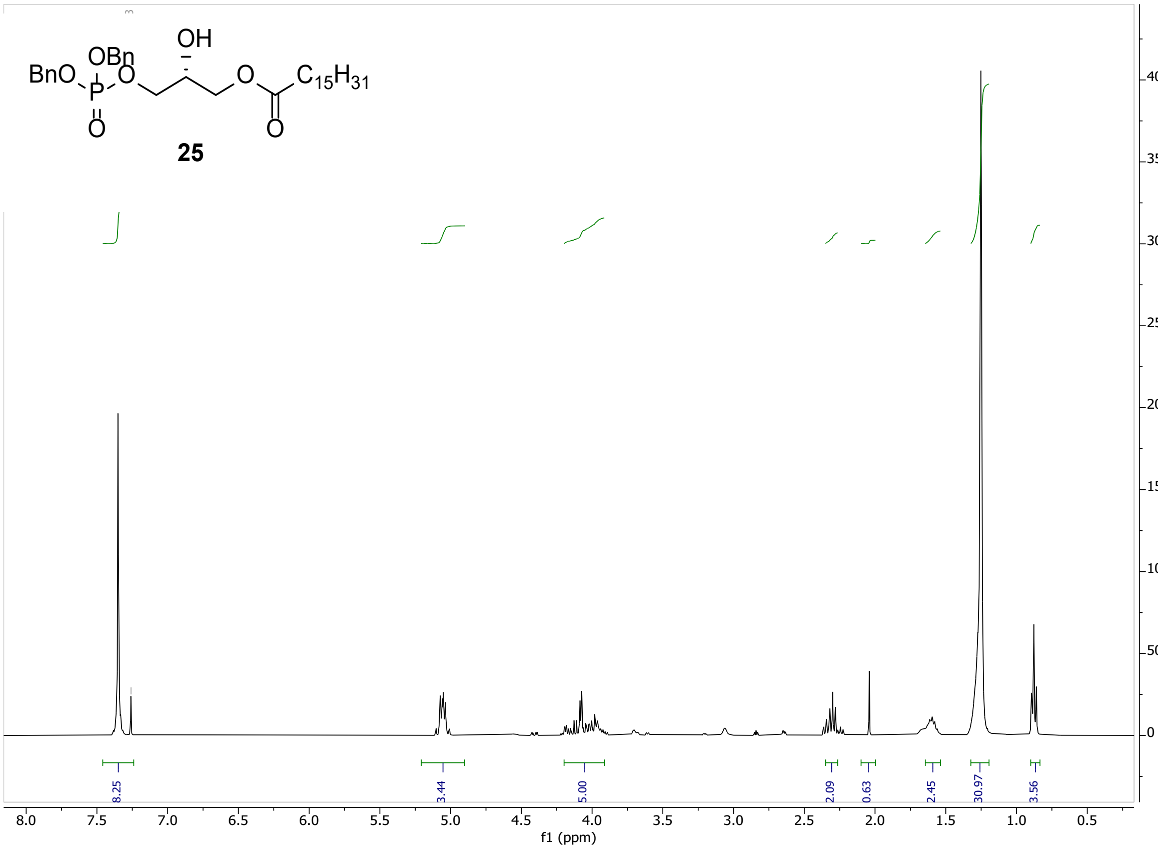
BnO
OBn
OH
C15H31
25
8.25
3.44
5.00
2.09
0.63
2.45
30.97
3.56
f1 (ppm)

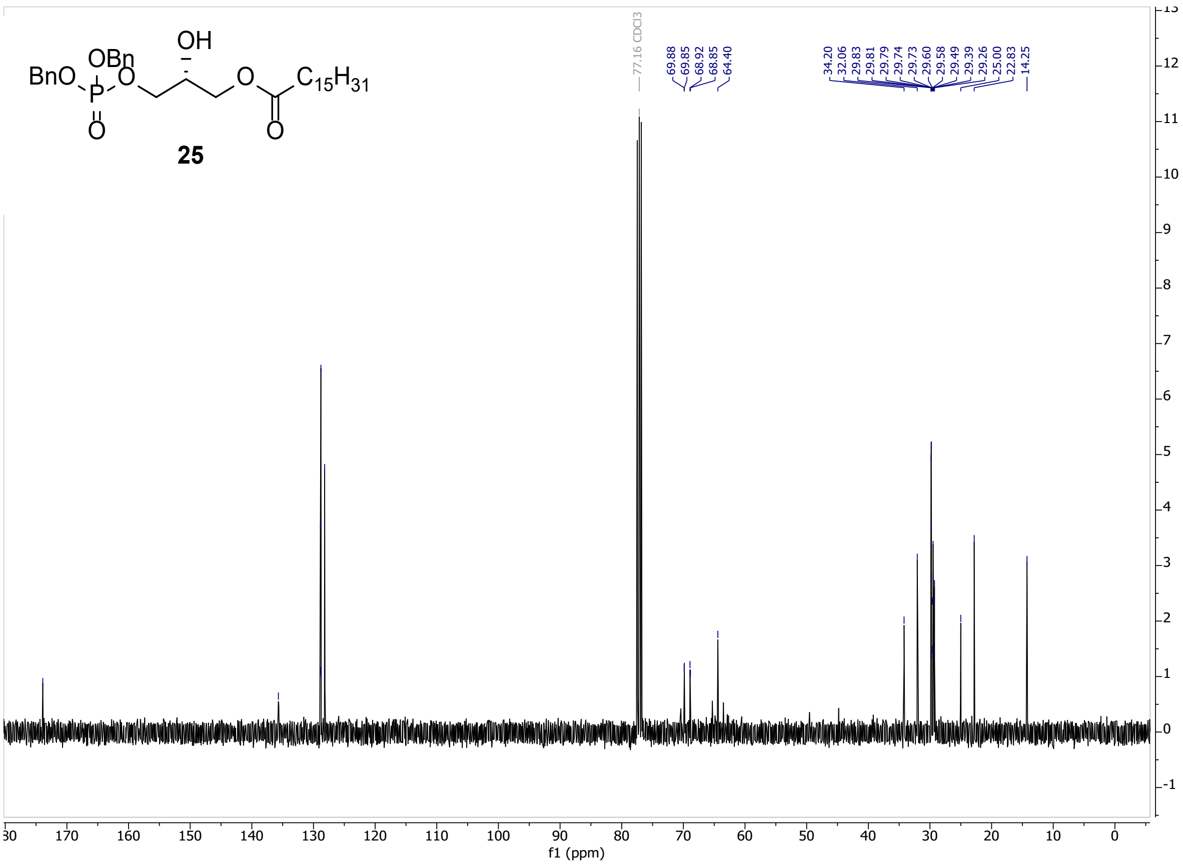
BnO
OBn
OH
C15H31
25
77.16 CDCl3
69.88
69.85
68.92
68.85
64.40
34.20
32.06
29.83
29.81
29.79
29.74
29.73
29.60
29.58
29.49
29.39
29.26
25.00
22.83
14.25
f1 (ppm)

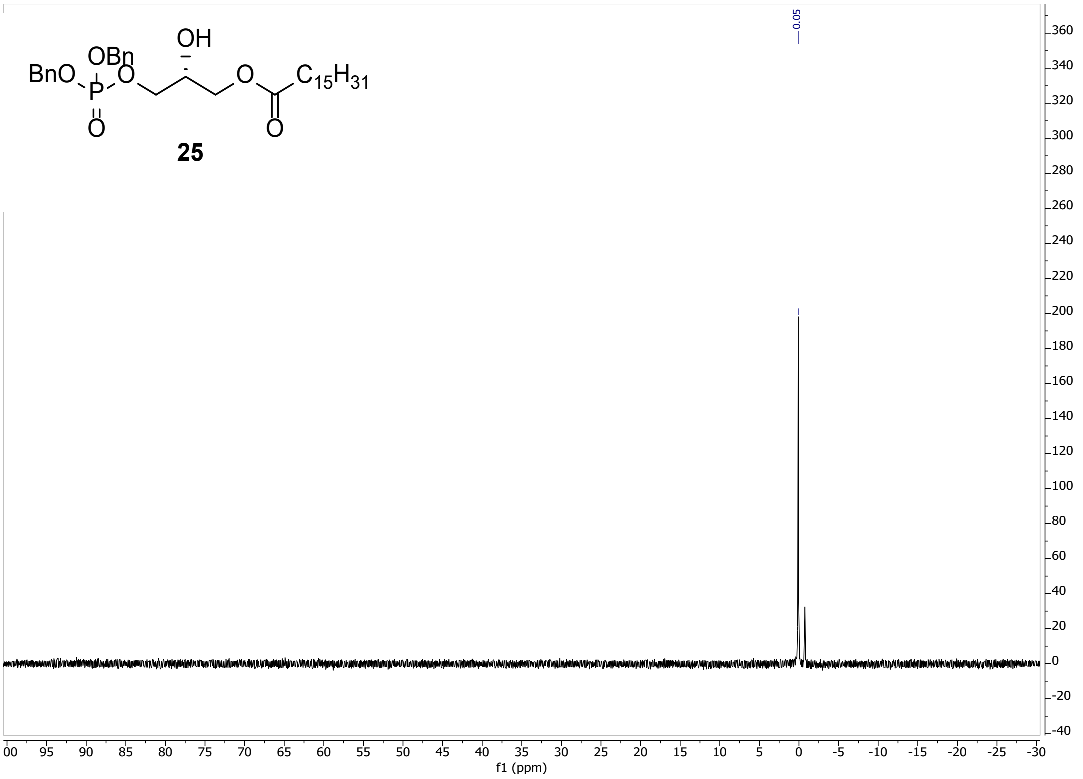
25
— 0.05
f1 (ppm)

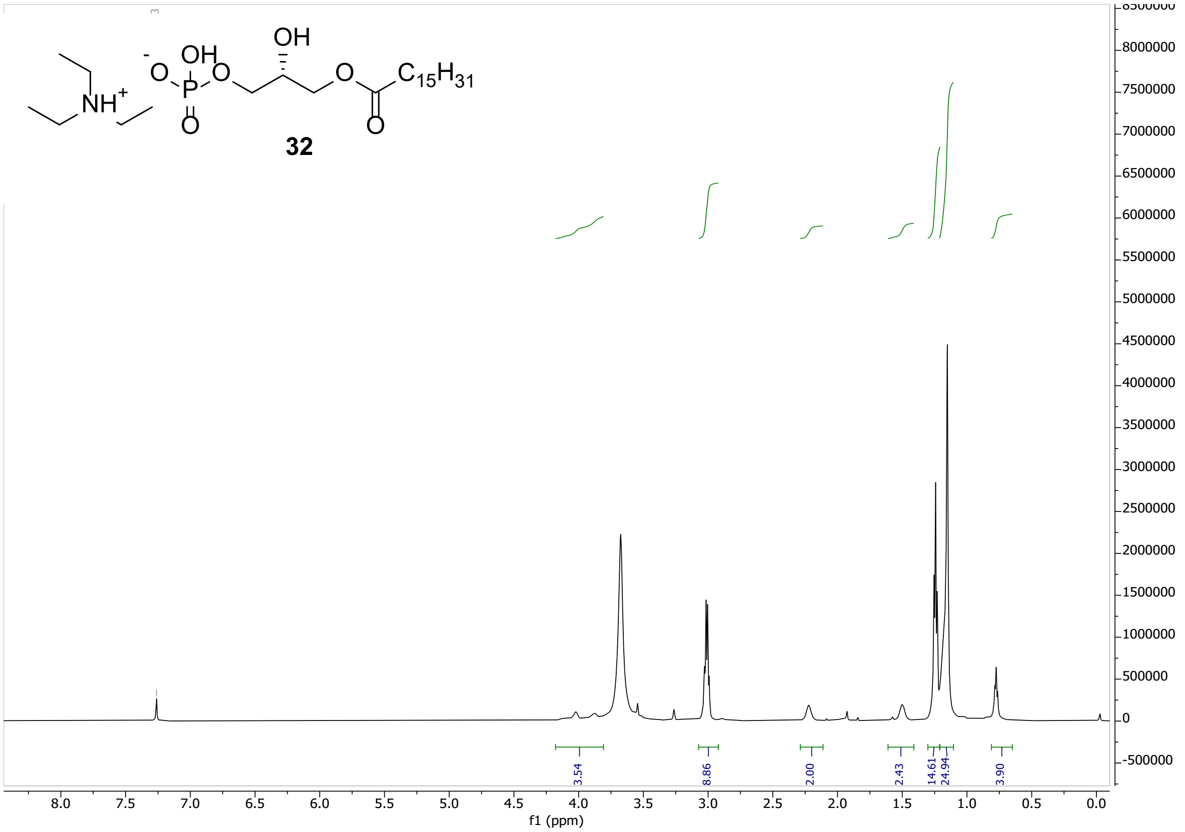

32

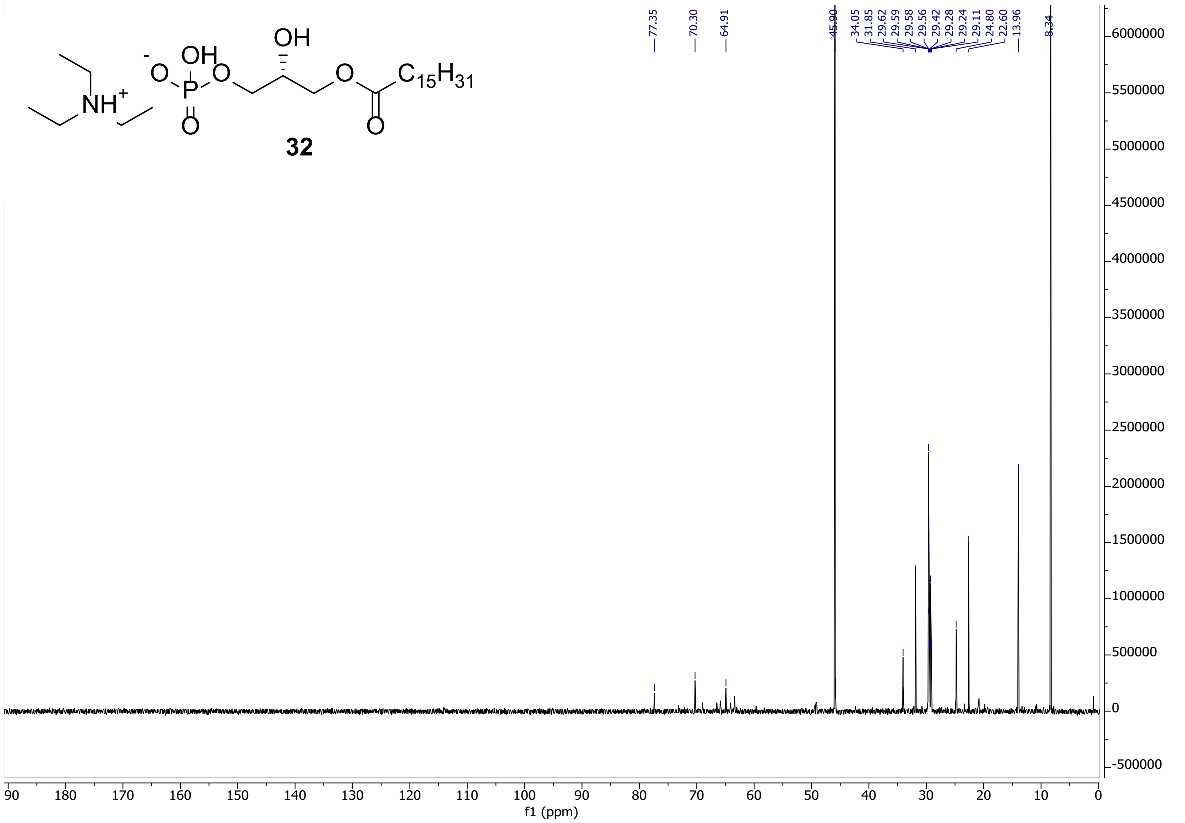

32

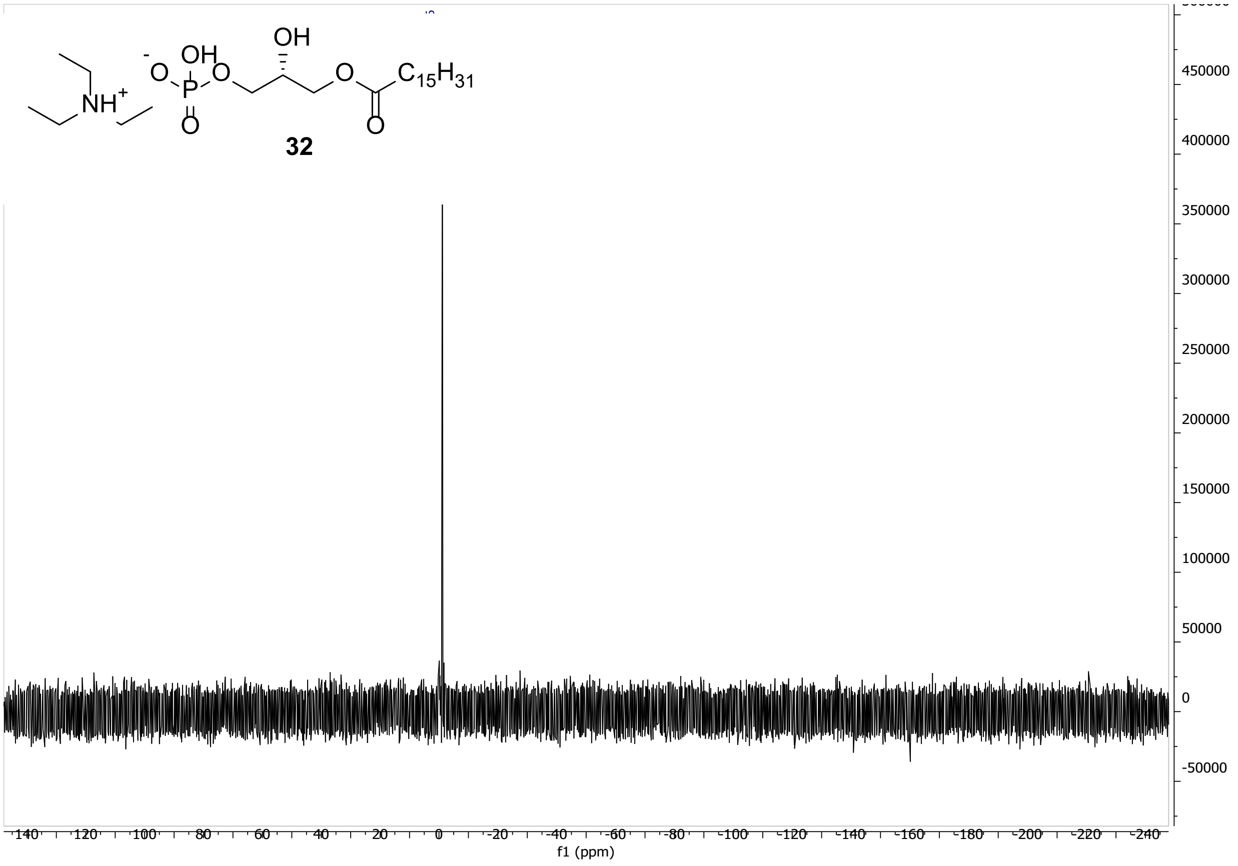
32
f1 (ppm)
450000
400000
350000
300000
250000
200000
150000
100000
50000
0
-50000

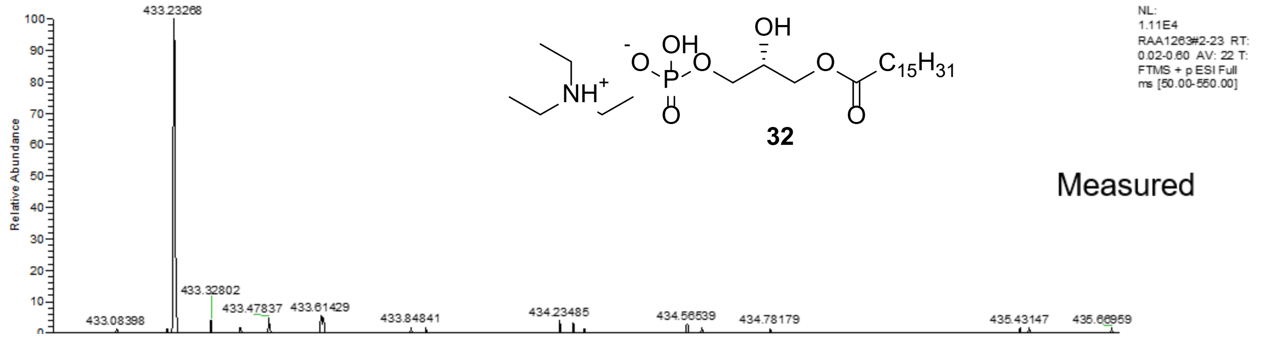
433.23268
433.32802
433.47837
433.61429
433.08398
433.84841
434.23485
434.56539
434.78179
435.43147
435.66959
Relative Abundance
NL:
1.11E4
RAA1263#2-23 RT:
0.02-0.60 AV: 22 T:
FTMS + p ESI Full
ms [50.00-550.00]
32
Measured

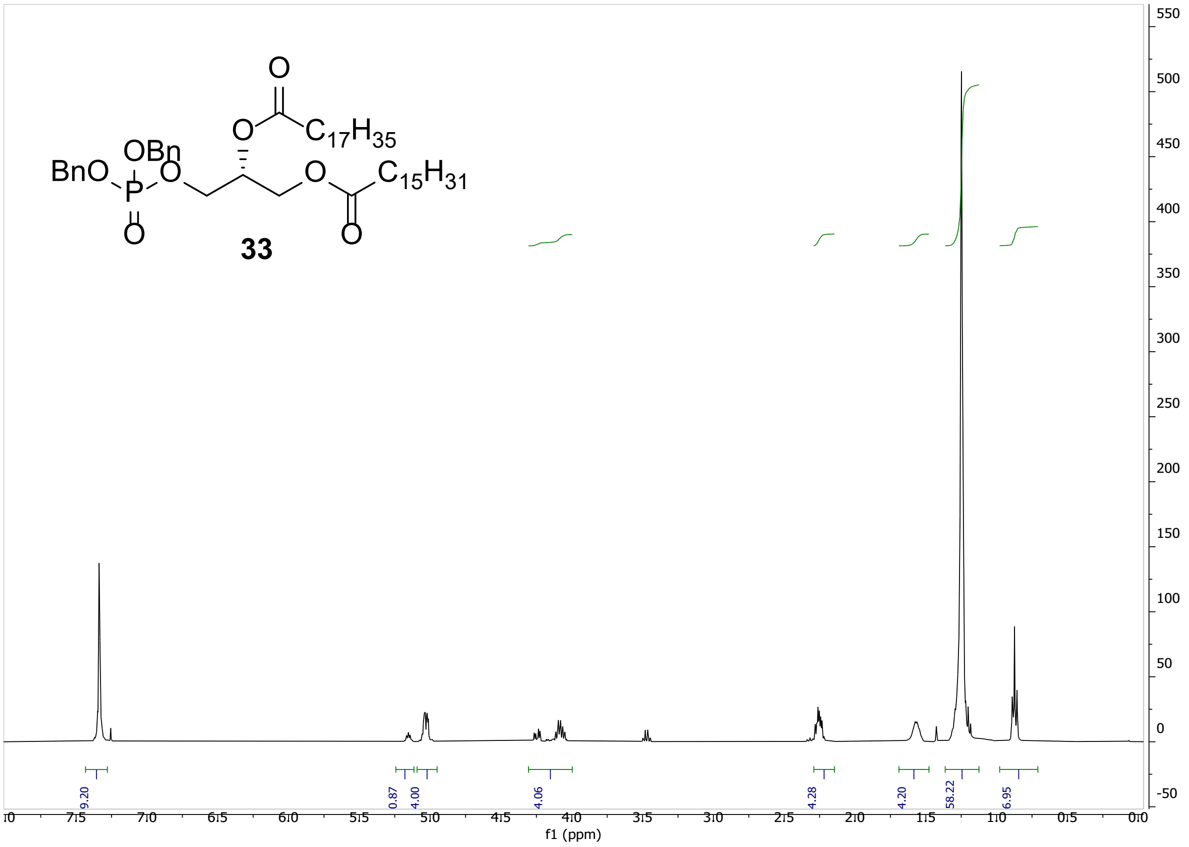
BnO
OBn
P
O
O
C17H35
C15H31
33
9.20
0.87
4.00
4.06
4.28
4.20
58.22
6.95
f1 (ppm)

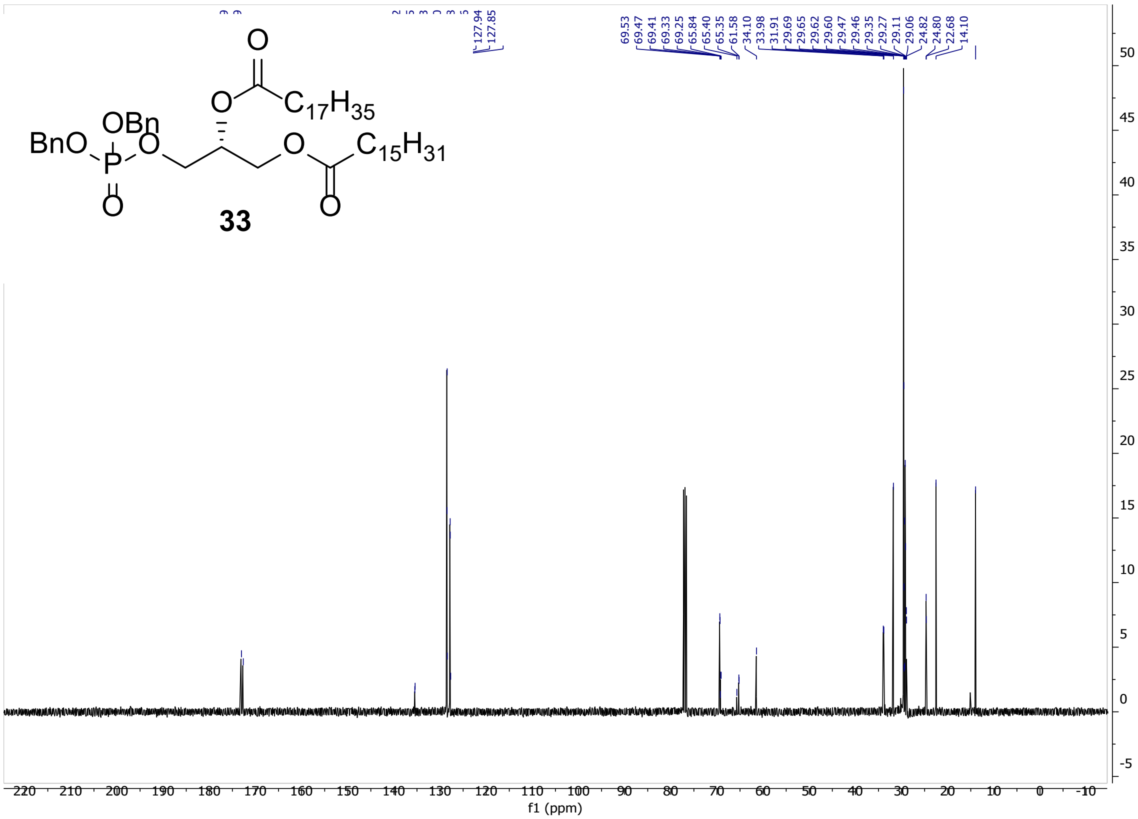
127.94
127.85
69.53
69.47
69.41
69.33
69.25
65.84
65.40
65.35
61.58
34.10
33.98
31.91
29.69
29.65
29.62
29.60
29.47
29.46
29.35
29.27
29.11
29.06
24.82
24.80
22.68
14.10
BnO
OBn
P
O
O
C17H35
C15H31
33
f1 (ppm)

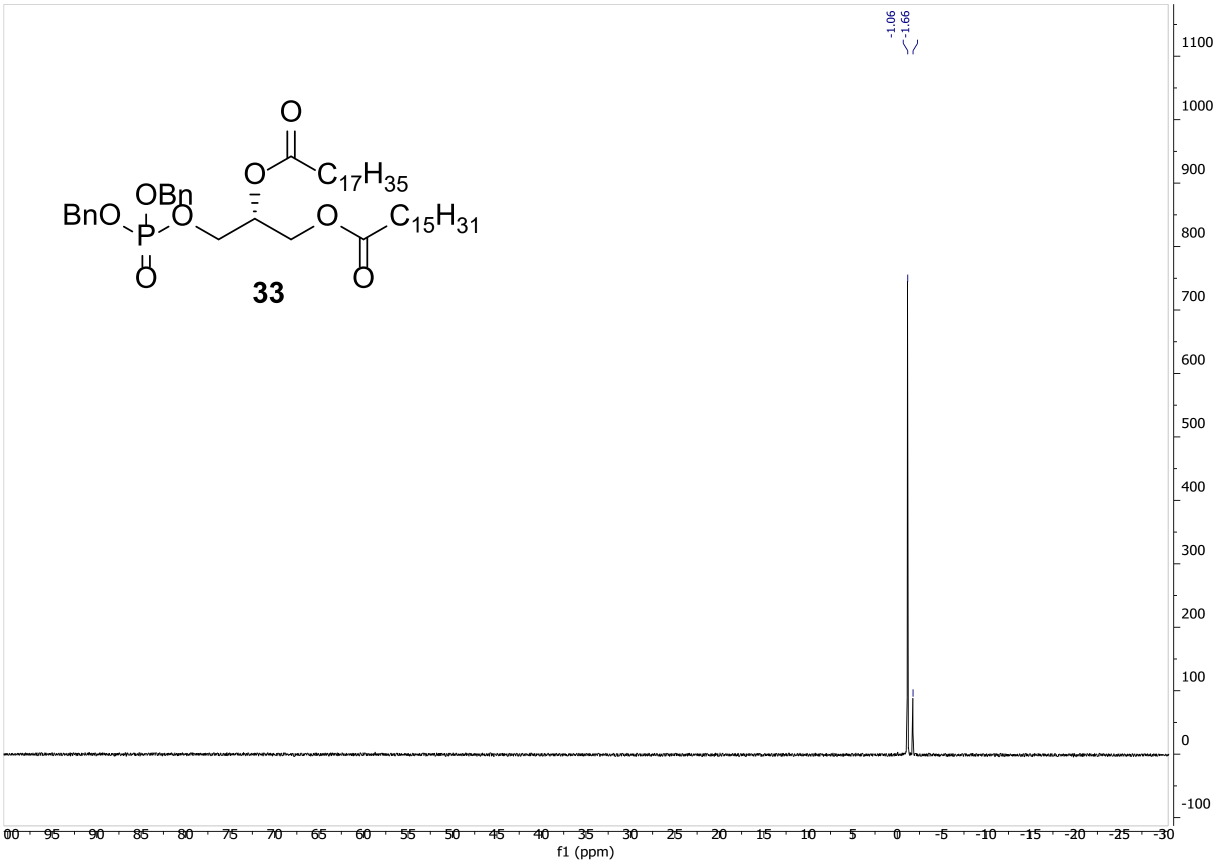

-1.06
-1.66
BnO
OBn
P
O
O
O
O
C17H35
O
C15H31
O
33
1100
1000
900
800
700
600
500
400
300
200
100
0
-100
00
95
90
85
80
75
70
65
60
55
50
45
40
35
30
25
20
15
10
5
0
-5
-10
-15
-20
-25
-30
f1 (ppm)

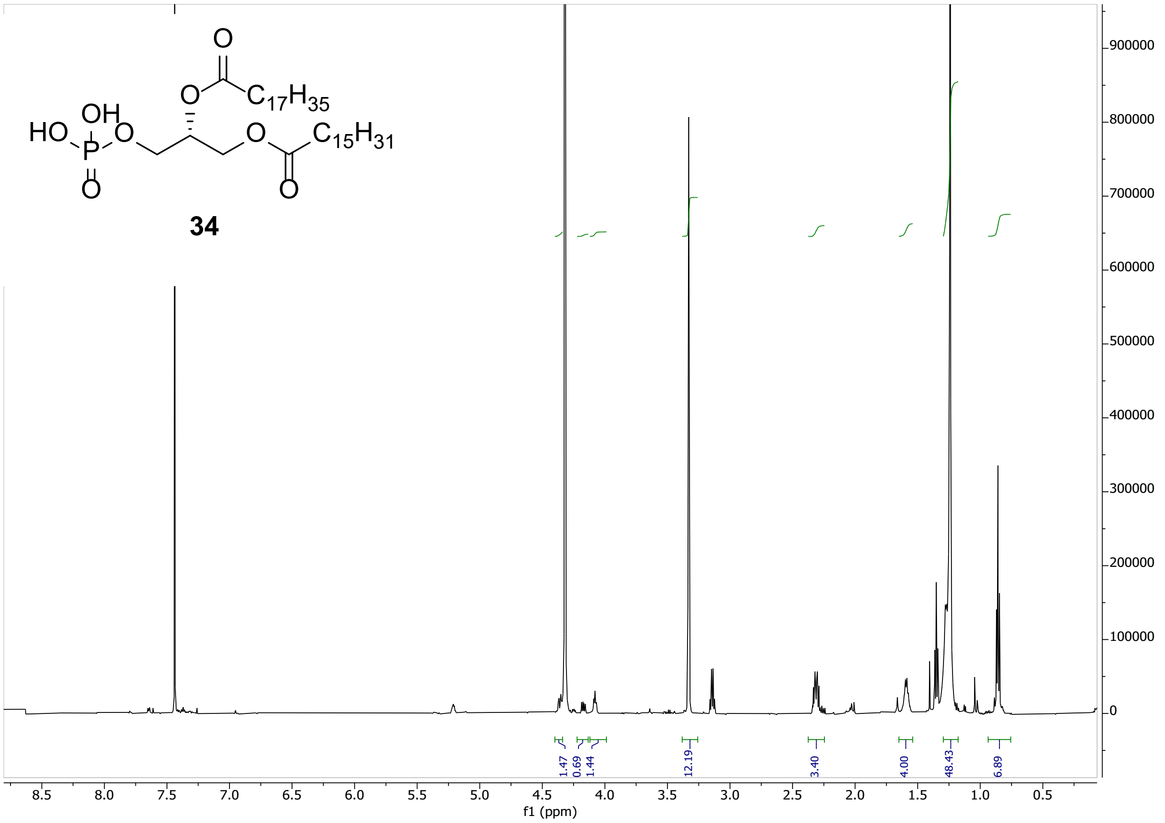
34
1.47
0.69
1.44
12.19
3.40
4.00
48.43
6.89
f1 (ppm)

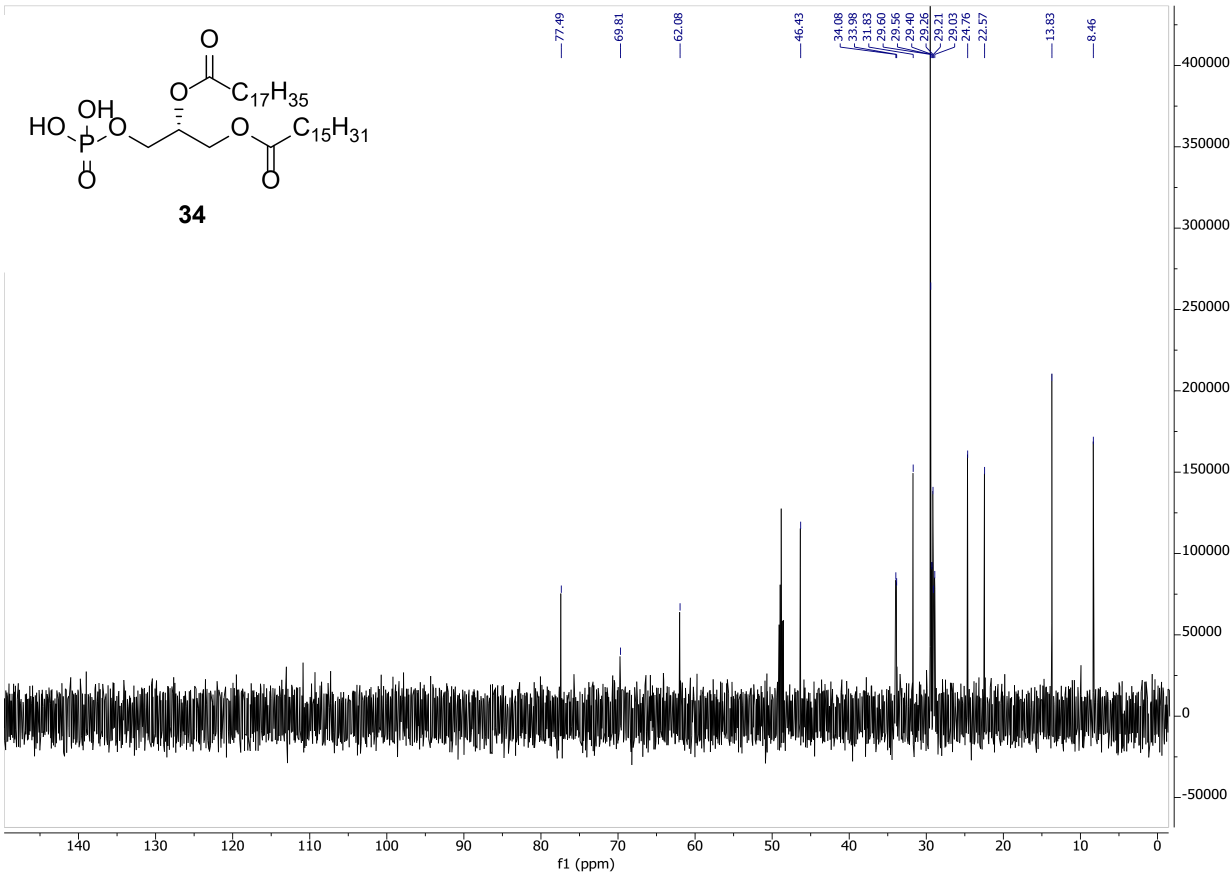
34
77.49
69.81
62.08
46.43
34.08
33.98
31.83
29.60
29.56
29.40
29.26
29.21
29.03
24.76
22.57
13.83
8.46
f1 (ppm)

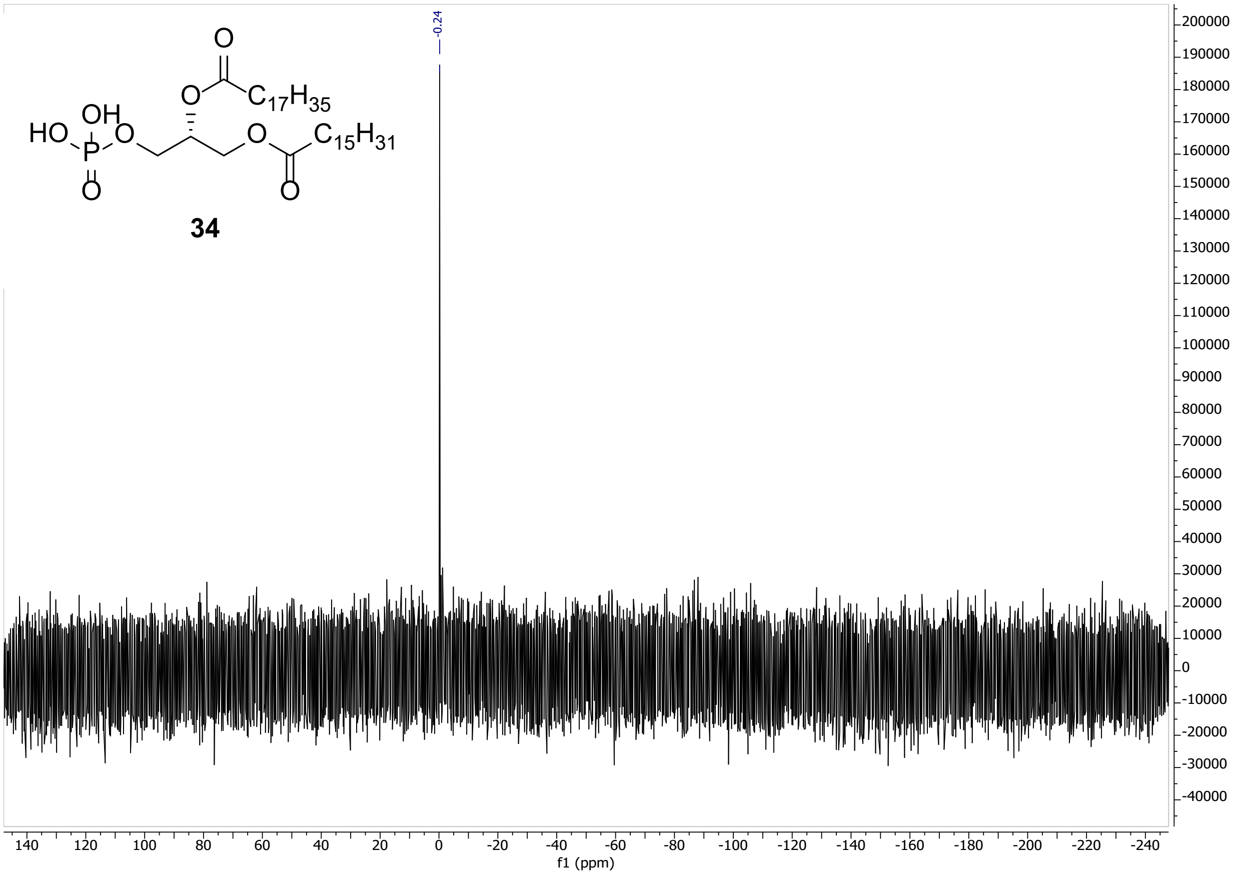
34
-0.24
f1 (ppm)

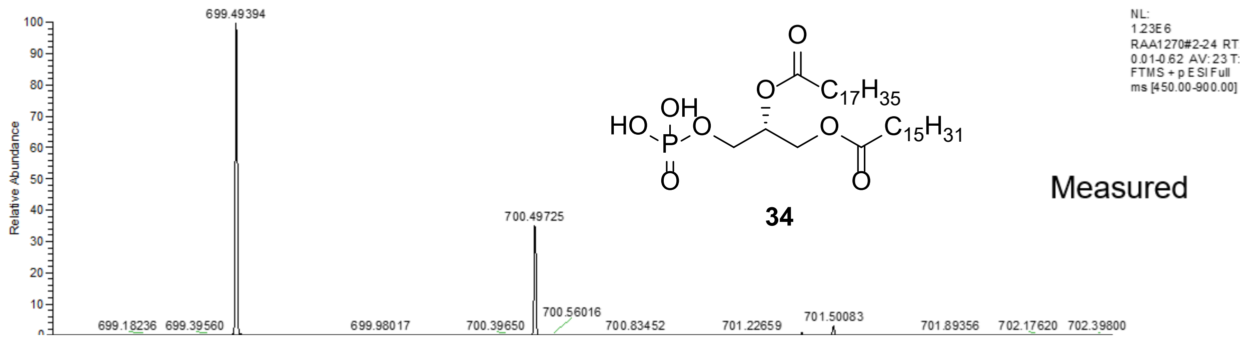
NL:
1.23E6
RAA1270#2-24 RT
0.01-0.62 AV: 23 T:
FTMS + p ESI Full
ms [450.00-900.00]
34
Measured
Relative Abundance
699.49394
700.49725
700.56016
701.50083

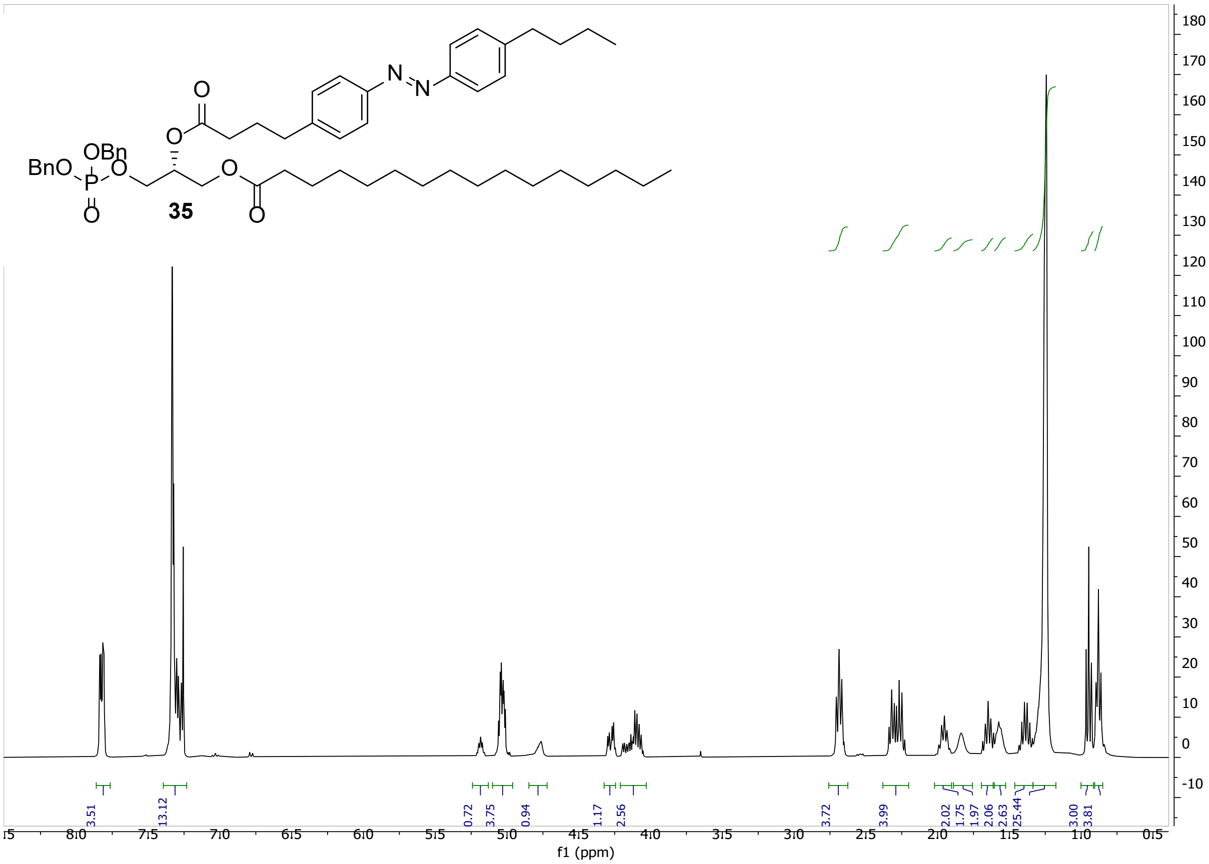
BnO
OBn
35
3.51
13.12
0.72
3.75
0.94
1.17
2.56
3.72
3.99
2.02
1.75
1.97
2.06
2.63
25.44
3.00
3.81
f1 (ppm)

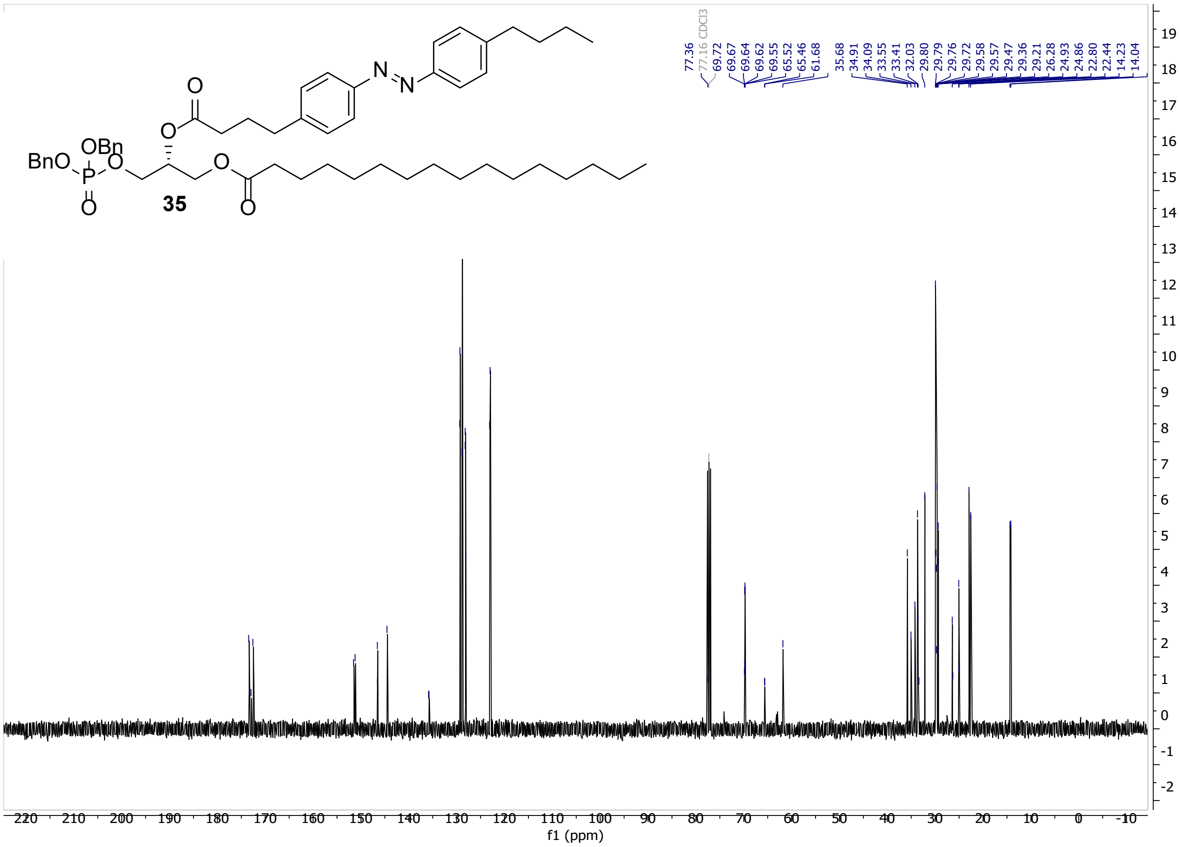
BnO
OBn
35
77.36
77.16 CDCl3
69.72
69.67
69.64
69.62
69.55
65.52
65.46
61.68
35.68
34.91
34.09
33.55
33.41
32.03
29.80
29.79
29.76
29.72
29.58
29.57
29.47
29.36
29.21
26.28
24.93
24.86
22.80
22.44
14.23
14.04
f1 (ppm)

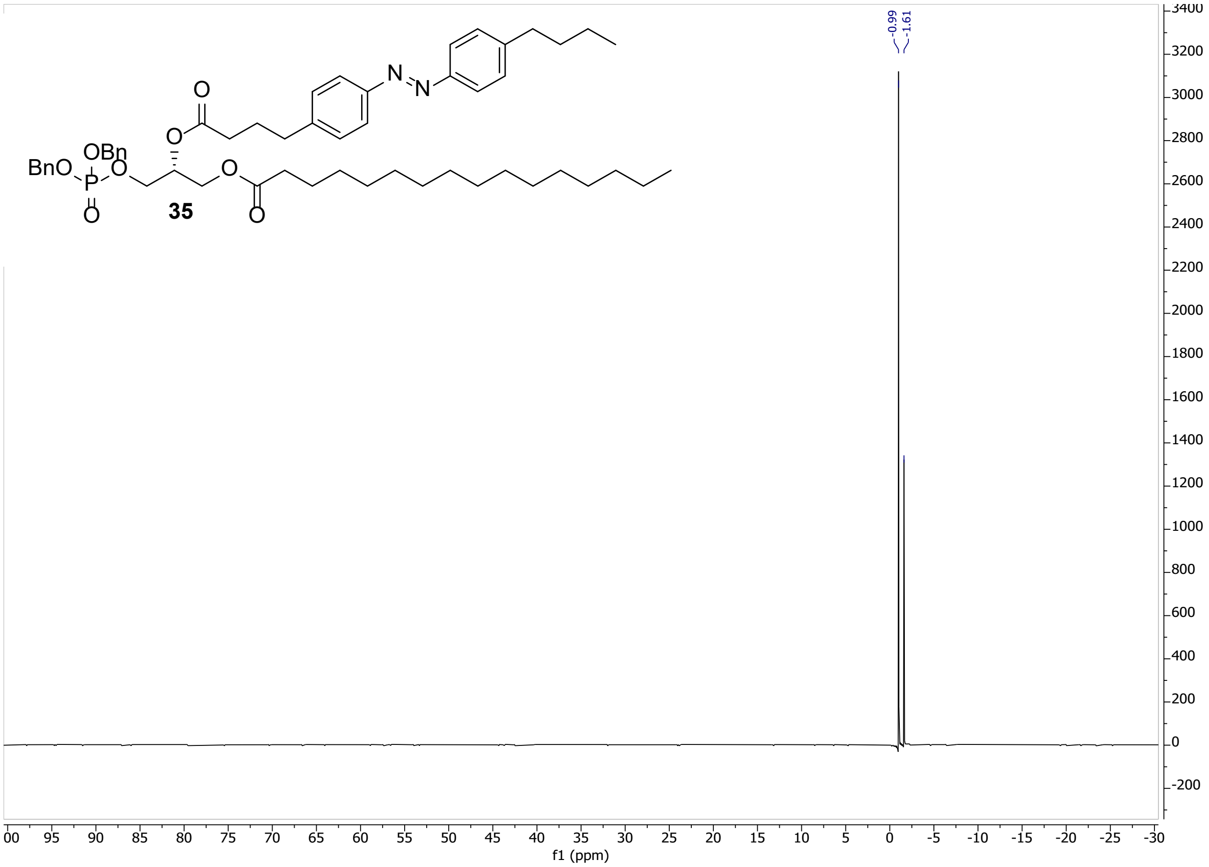
-0.99
-1.61
BnO
OBn
P
O
35
N
N
f1 (ppm)

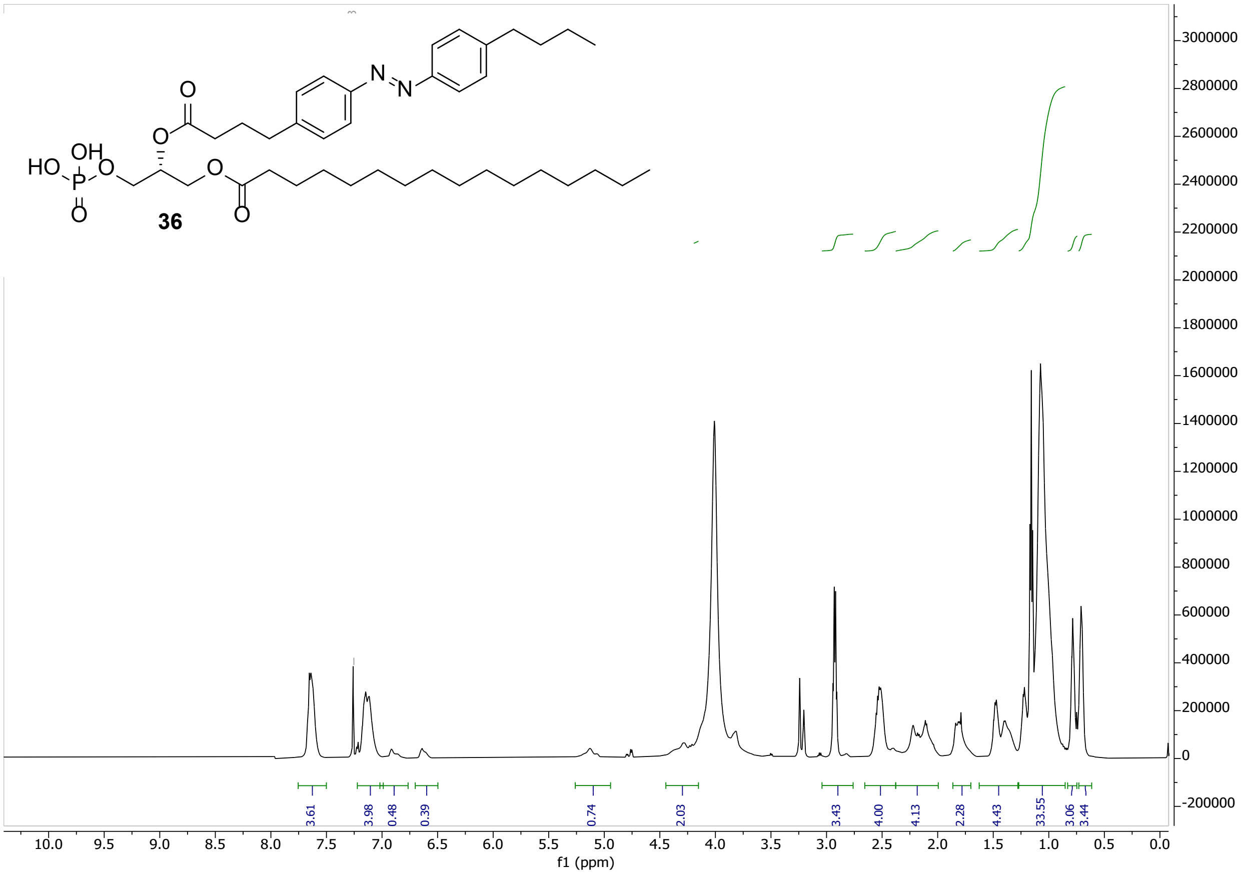

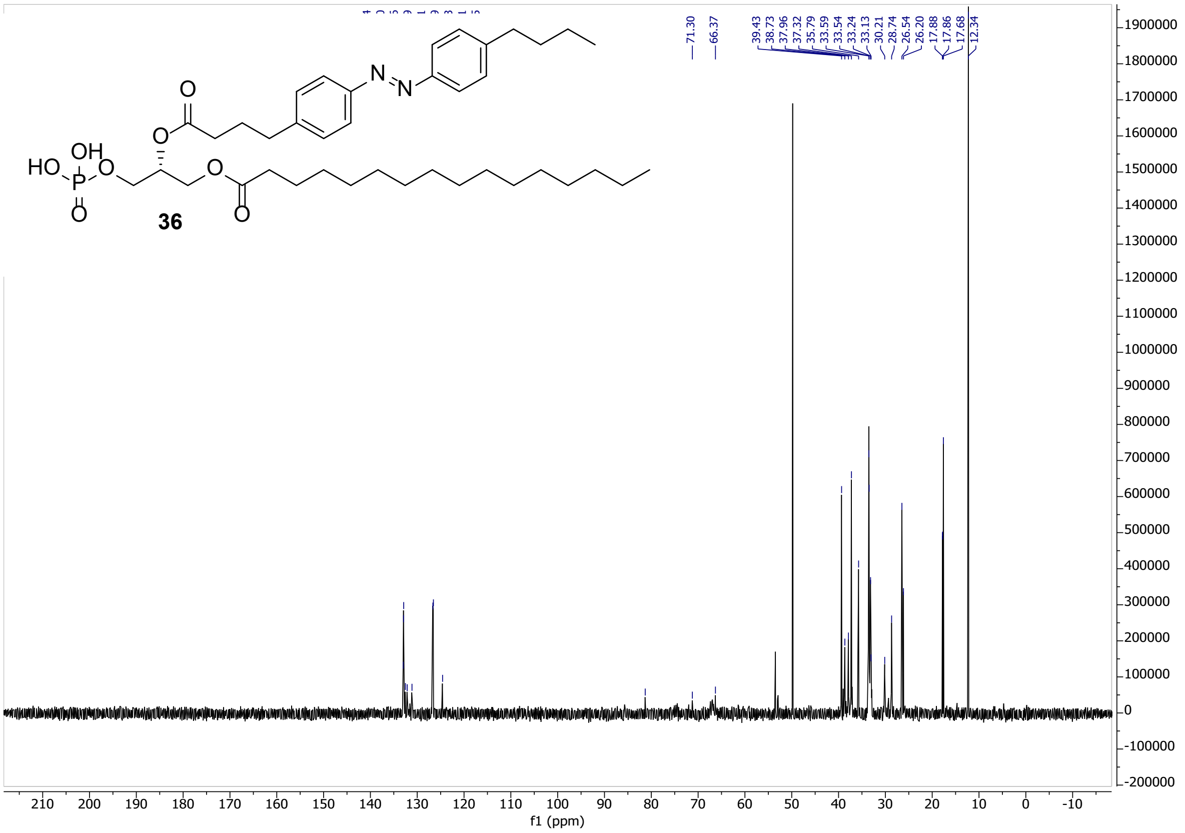

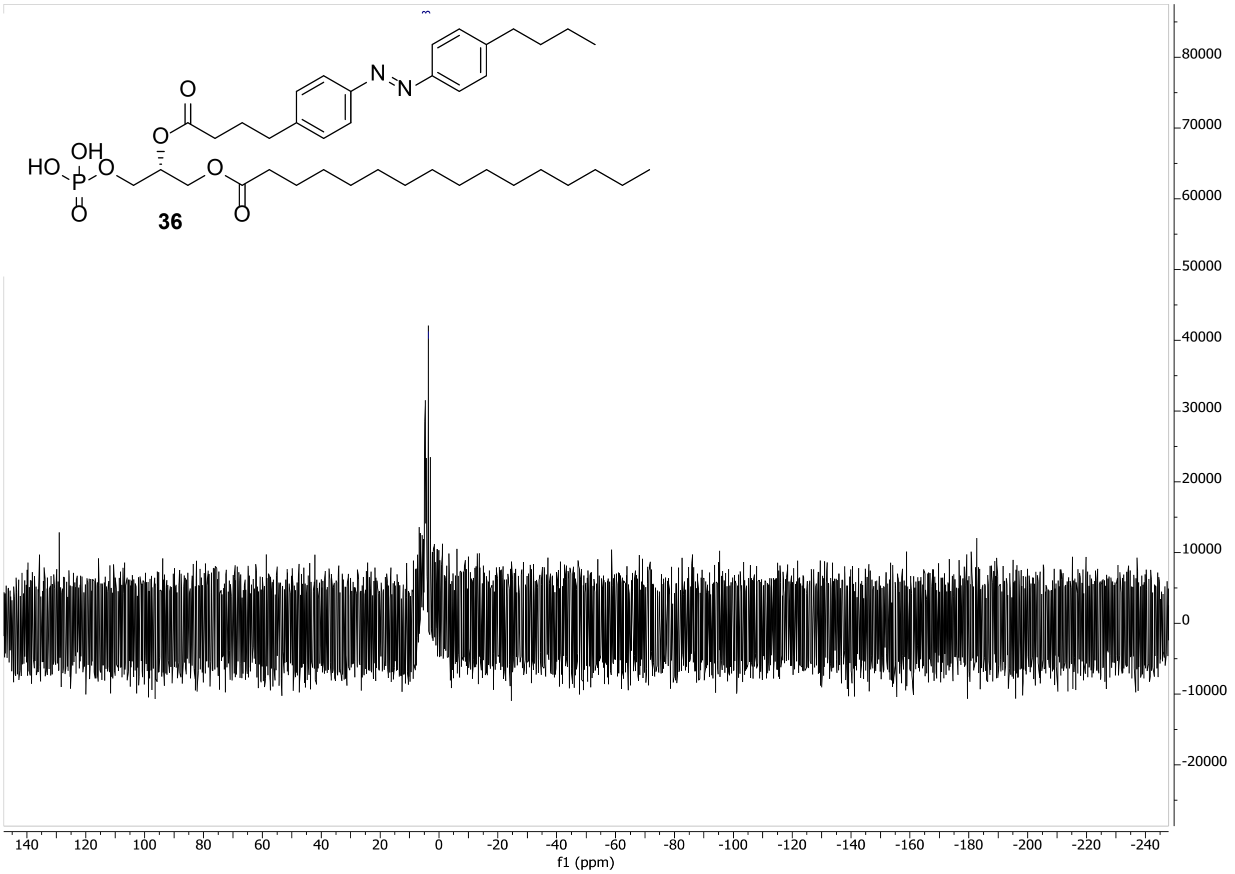

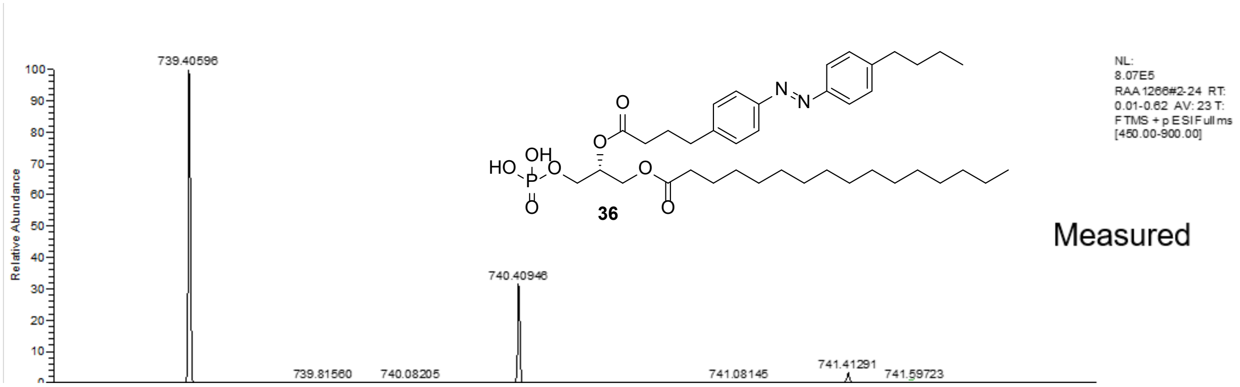

References

[1] Meyer, O.; Ponaire, S.; Rohmer, M.; Grosdemange-Billiard, C. Lewis Acid Mediated Regioselective Ring Opening of Benzylglycidol with Dibenzyl Phosphate: Short and Attractive Synthesis of Dihydroxyacetone Phosphate. *Org. Lett.* **2006**, *8* (19), 4347–4350.
